# Dual Inhibition of CDK4/6 and CDK7 Suppresses Triple‐Negative Breast Cancer Progression via Epigenetic Modulation of SREBP1‐Regulated Cholesterol Metabolism

**DOI:** 10.1002/advs.202413103

**Published:** 2024-12-10

**Authors:** Yilan Yang, Jiatao Liao, Zhe Pan, Jin Meng, Li Zhang, Wei Shi, Xiaofang Wang, Xiaomeng Zhang, Zhirui Zhou, Jurui Luo, Xingxing Chen, Zhaozhi Yang, Xin Mei, Jinli Ma, Zhen Zhang, Yi‐Zhou Jiang, Zhi‐Min Shao, Fei Xavier Chen, Xiaoli Yu, Xiaomao Guo

**Affiliations:** ^1^ Department of Radiation Oncology Fudan University Shanghai Cancer Center No.270 Dong'an Road Shanghai 200032 China; ^2^ Department of Oncology Shanghai Medical College Fudan University No.270 Dong'an Road Shanghai 200032 China; ^3^ Shanghai Clinical Research Center for Radiation Oncology No.270 Dong'an Road Shanghai 200032 China; ^4^ Shanghai Key Laboratory of Radiation Oncology No.270 Dong'an Road Shanghai 200032 China; ^5^ Radiation Oncology Center Huashan Hospital No.12 Wulumuqi Middle Road Shanghai 200040 China; ^6^ Department of Radiation Oncology Renji Hospital School of Medicine Shanghai Jiao Tong University No.1630 Dongfang Road Shanghai 200127 China; ^7^ Department of Breast Surgery Fudan University Shanghai Cancer Center No. 270 Dong'an Road Shanghai 200032 China; ^8^ Fudan University Shanghai Cancer Center Institutes of Biomedical Sciences State Key Laboratory of Genetic Engineering Shanghai Key Laboratory of Medical Epigenetics Shanghai Key Laboratory of Radiation Oncology Fudan University No.131 Dong'an Road Shanghai 200032 China

**Keywords:** breast cancer, CDK4/6, CDK7, cholesterol metabolism

## Abstract

Inhibitors targeting cyclin‐dependent kinases 4 and 6 (CDK4/6) to block cell cycle progression have been effective in treating hormone receptor‐positive breast cancer, but triple‐negative breast cancer (TNBC) remains largely resistant, limiting their clinical applicability. The study reveals that transcription regulator cyclin‐dependent kinase7 (CDK7) is a promising target to circumvent TNBC's inherent resistance to CDK4/6 inhibitors. Combining CDK4/6 and CDK7 inhibitors significantly enhances therapeutic effectiveness, leading to a marked decrease in cholesterol biosynthesis within cells. This effect is achieved through reduced activity of the transcription factor forkhead box M1 (FOXM1), which normally increases cholesterol production by inducing SREBF1 expression. Furthermore, this dual inhibition strategy attenuates the recruitment of sterol regulatory element binding transcription factor 1 (SREBP1) and p300 to genes essential for cholesterol synthesis, thus hindering tumor growth. This research is corroborated by an in‐house cohort showing lower survival rates in TNBC patients with higher cholesterol production gene activity. This suggests a new treatment approach for TNBC by simultaneously targeting CDK4/6 and CDK7, warranting additional clinical trials.

## Graphical abstract

A novel approach is introduced to overcome the intrinsic resistance of TNBC to CDK4/6 inhibitors through targeting the transcription regulator CDK7. This dual inhibition strategy induces synthetic lethality in multiple TNBC models, both in vitro and in vivo, opening a pioneering therapeutic avenue for TNBC treatment.

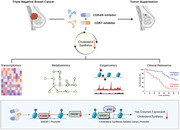

## 1 Introduction

Triple‐negative breast cancer (TNBC) comprises ≈10–20% of breast cancer cases and is characterized by the absence of estrogen receptor (ER) expression, progesterone receptor (PR) expression, and human epidermal growth factor receptor 2 (HER2) amplification.^[^ ^1^ ^]^ Compared to other subtypes, TNBC exhibits clinical characteristics of earlier onset, higher metastatic potential, and worse clinical outcomes.^[^ ^2^ ^]^ Due to the limited progress in targeted therapies, chemotherapy remains the predominant treatment for TNBC patients. However, only ≈35% of TNBC patients benefit from chemotherapy,^[^ ^2^ ^]^ making the development of novel therapeutic strategies an urgent priority for TNBC treatment.

Cyclin‐dependent kinase 7 (CDK7), a key regulator of cell cycle progression and gene transcription, has emerged as a promising target in TNBC.^[^ ^3^, ^4^ ^]^ CDK7 inhibitors, including THZ1, disrupt the activities of cell cycle‐associated CDKs (CDK1, 2, 4, and 6) and suppress the phosphorylation of the C‐terminal domain of RNA polymerase II, exhibiting anti‐tumor effects in preclinical TNBC models.^[^ ^5^ ^]^ However, there are no ongoing or completed clinical trials for THZ1, possibly due to the potent CDK12/13 off‐target effects upon THZ1 administration.^[^ ^6^, ^7^ ^]^ To address this limitation, novel CDK7 inhibitors, such as YKL‐5‐124,^[^ ^8^ ^]^ CT7001 (also known as Samuraciclib),^[^ ^9^ ^]^ and SY‐1365,^[^ ^10^ ^]^ have been developed to achieve better selectivity. Encouragingly, several of these compounds have progressed to phase I/II clinical trials. Current research on highly selective CDK7 inhibitors is predominantly in pan‐cancer contexts, leaving the role and underlying mechanisms in TNBC relatively unexplored.

The oral cyclin‐dependent kinases 4 and 6 (CDK4/6) inhibitors, palbociclib, ribociclib, and abemaciclib, have significantly improved the progression‐free survival of hormone receptor (HR)‐positive, HER2‐negative (HR+/HER2‐) metastatic or advanced breast cancer patients when combined with endocrine therapy.^[^ ^11^, ^12^, ^13^, ^14^, ^15^, ^16^ ^]^ The therapeutic landscape further expanded with the monarchE trial,^[^ ^17^, ^18^ ^]^ in which abemaciclib demonstrated efficacy not only in metastatic settings but also in HR‐positive, high‐risk early‐stage breast cancer. Consequently, the primary focus of clinical trials involving CDK4/6 inhibitors has been on HR‐positive breast cancer patients, while the exploration of these agents in TNBC patients remains limited. In preclinical studies, there is a growing emphasis on overcoming the intrinsic resistance of TNBC to CDK4/6 inhibitors.^[^ ^19^ ^]^ Various agents,^[^ ^20^, ^21^, ^22^, ^23^, ^24^, ^25^, ^26^, ^27^ ^]^ including PI3K inhibitors,^[^ ^20^, ^25^ ^]^ BET inhibitors,^[^ ^22^, ^24^ ^]^ and lysosome degradation compounds,^[^ ^21^ ^]^ have shown promise in synergizing with CDK4/6 inhibitors in TNBC. This underscores the combination therapy as an appealing strategy to counteract intrinsic resistance. Here, we identified a novel target, CDK7, which impacts the responsiveness of TNBC to CDK4/6 inhibition. Our findings further validated the synergistic lethality of co‐inhibiting CDK7 and CDK4/6 in TNBC, providing a potential treatment avenue for TNBC patients.

## 2 Results

### 2.1 CDK7 Inhibition Upregulates Luminal Gene Signatures and Renders TNBC More Sensitive to Abemaciclib

**Figure 1:**
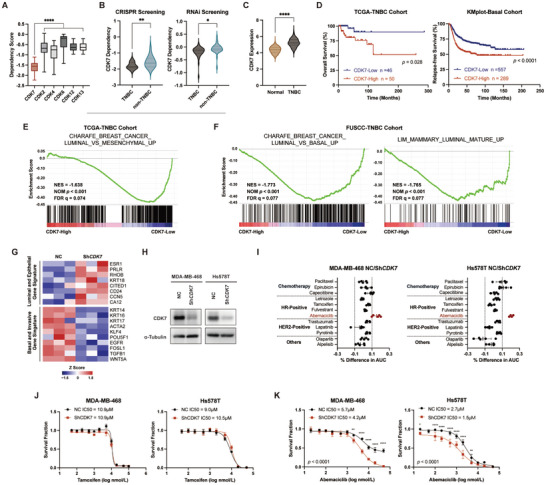
CDK7 inhibition elevates luminal‐related transcriptional activities and renders TNBC more sensitive to abemaciclib. A) The box plots depicting the dependency score of CDK7 and several other CDKs in TNBC cells (*n* = 25) using CRISPR screening datasets from the Broad Institute DepMap portal. *p* values were calculated using one‐way ANOVA, ****p < 0.0001. B) Violin plots of CDK7 dependency scores in TNBC and non‐TNBC breast cancer cells (*n* = 24 for TNBC, *n* = 17 for non‐TNBC) using CRISPR and RNAi screening datasets from the Broad Institute DepMap portal. *p* values were calculated using unpaired *t*‐test, *p < 0.05,**p < 0.01. C) Violin plots of CDK7 mRNA expression levels in the TCGA‐TNBC dataset (*n* = 140) and normal breast tissues from the GTEx dataset (*n* = 459). P values were calculated using an unpaired *t*‐test, ****p < 0.0001. D) Kaplan–Meier plots of CDK7 expression in TNBC patients using the TCGA cohort and KMplot cohort. Data were analyzed using the log‐rank test. E,F) Gene set enrichment analysis (GSEA) of RNA‐Seq data for CDK7‐high and CDK7‐low patients in the TCGA‐TNBC (E) and FUSCC‐TNBC (F) cohorts. NES, normalized enrichment score, NOM, nominal, FDR, false discovery rate. G) Heatmap summarizing the RNA‐Seq data of selected luminal/epithelial marker genes and basal/invasive marker genes in normal control (NC) and CDK7‐knockdown (ShCDK7) Hs578T cells (*n* = 3). H) Immunoblot validation of CDK7 knockdown in MDA‐MB‐468 and Hs578T cells. I) Differences in drug sensitivities between MDA‐MB‐468‐NC and MDA‐MB‐468‐ShCDK7 cells, as well as between Hs578T‐NC and Hs578T‐ShCDK7 cells. Data are mean ± SD of 5 replicates. J,K) Dose‐response curves of tamoxifen (J) and abemaciclib (K) between NC and ShCDK7 MDA‐MB‐468 and Hs578T cells. Data are mean ± SD of 3–5 experimental replicates. *p* values were analyzed using a two‐way ANOVA test with Bonferroni correction.

Previous work demonstrated that TNBC cell lines, but not HR‐positive breast cancer cell lines, required CDK7 for proliferation.^[^ ^5^ ^]^ Using DepMap CRISPR screening datasets, we discovered that CDK7 demonstrated stronger dependency scores compared to other CDKs in TNBC (**Figure** **1**A). Furthermore, TNBC cells showed a higher reliance on CDK7 compared to non‐TNBC breast cancer cells (Figure 1B). Analysis of TCGA and GTEx databases revealed that CDK7 mRNA expression is the lowest among the four breast cancer subtypes (Figure S1A, Supporting Information), however, it remained significantly higher than that in normal breast tissues (Figure 1C). TNBC patients with higher CDK7 expression exhibited worse overall survival (OS), relapse‐free survival (RFS), and distant metastasis‐free survival (DMFS) (Figure 1D; Figure S1B, Supporting Information). However, CDK7 expression did not exhibit prognostic significance in the remaining breast cancer subtypes (Figure S1C, Supporting Information), indicating the prognostic value of CDK7 may be restricted to TNBC. To explore the underlying mechanisms between CDK7 and clinical outcomes, The Cancer Genome Atlas (TCGA) and Fudan University Shanghai Cancer Center (FUSCC) TNBC patients were stratified based on CDK7 expression. Intriguingly, gene set enrichment analysis (GSEA) revealed upregulation of luminal gene signatures in CDK7‐low subsets in both cohorts (Figure 1E,F). Similar increases in luminal transcription activities occurred upon CDK7 knockdown (ShCDK7) in Hs578T cells (Figure 1G; Figure S1D, Supporting Information).

These findings suggest that CDK7 suppression may induce a more differentiated, HR‐positive‐like state in TNBC. Therefore, we hypothesized that CDK7 expression might impact the susceptibility to endocrine therapy and CDK4/6 inhibitors, which are commonly used in HR‐positive patients. Subsequently, we carried out a cellular viability screen in normal control (NC) and ShCDK7 TNBC cells (Figure 1H), using 12 clinically used agents targeting various subtypes. Our observations validated a higher abemaciclib sensitivity in ShCDK7 cells, while the sensitivities to other agents exhibited no substantive changes across ShCDK7 and NC cells (Figure 1I; Figure S1E,F, Supporting Information). To confirm the specificity of CDK7's impact on CDK4/6 inhibitors, but not other endocrine therapeutics, we generated additional NC/ShCDK7 TNBC cells (Figure S1G, Supporting Information). Utilizing a panel of constructed TNBC cell lines, we further confirmed hypersensitivity to abemaciclib upon CDK7 knockdown, whereas no changes in tamoxifen response (Figure 1J,K; Figure S1H,I, Supporting Information). Thus, CDK7 suppression selectively sensitized TNBC cells to CDK4/6 inhibition.

### 2.2 The synergistic Lethality of Combination Treatments In Vitro and In Vivo

**Figure 2:**
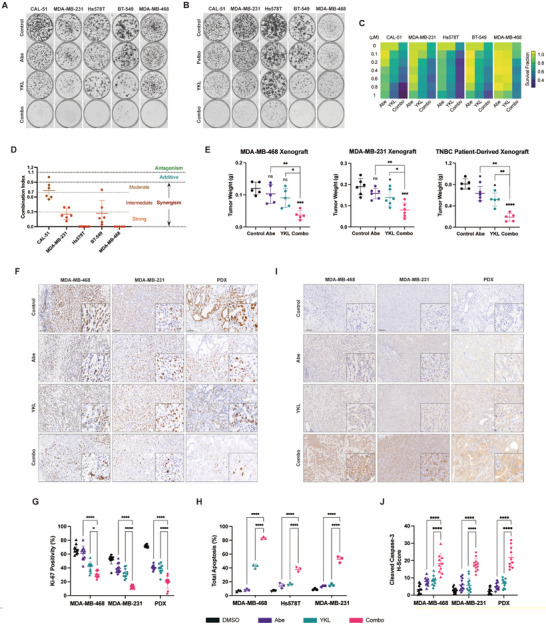
The synergistic lethality of co‐inhibiting CDK4/6 and CDK7 in TNBC. A) Colony formation images of TNBC cells following a 48 h exposure to the combination of abemaciclib with YKL‐5‐124. Representative images from 3 biological replicates are provided. B) Colony formation images of TNBC cells following a 48 h exposure to the combination of palbociclib with YKL‐5‐124. Representative images from 3 biological replicates are provided. C) Heatmap of survival fractions in TNBC cells after 48 h exposure to gradient concentrations of abemaciclib, YKL‐5‐124, and the combined treatment (abemaciclib at gradient concentrations with YKL‐5‐124 at fixed concentrations). Data are shown as mean (*n* = 3 biological replicates). D) Combination index values for TNBC cells treated with abemaciclib plus YKL‐5‐124, calculated by CompuSyn software. Data are represented as mean ± SD. E) Tumor weights of MDA‐MB‐468 and patient‐derived xenografts (PDX) after 21 days of treatment with control, abemaciclib (50 mg kg^−1^), YKL‐5‐124 (2 mg kg^−1^), or the combination (*n* = 5). Tumor weights of MDA‐MB‐231 xenografts after 14 days of treatment with control, abemaciclib (50 mg kg^−1^), YKL‐5‐124 (5 mg kg^−1^), or the combination (*n* = 6). Data are shown as mean ± SD. *p* values were calculated using one‐way ANOVA, *p < 0.05, **p < 0.01, ***p < 0.001, ****p < 0.0001. F) Immunohistochemistry (IHC) staining of ki‐67 in tumor sections of MDA‐MB‐468, MDA‐MB‐231, and patient‐derived xenografts. Scale bar, 100 µm. G) Quantifications of ki‐67 staining in tumor sections of MDA‐MB‐468 (*n* = 10), MDA‐MB‐231 (*n* = 12), and patient‐derived xenografts (*n* = 10). Two representative images per tumor were used to quantify the ki‐67 positivity. Data are represented as mean ± SD. *p* values were calculated using one‐way ANOVA, *p < 0.05, ****p < 0.0001. H) Percentage of total apoptotic cells after 96 h of treatment with abemaciclib, YKL‐5‐124, and the combination (*n* = 3). Data are presented as mean ± SD. *p* values were calculated using one‐way ANOVA, ****p < 0.0001. I) IHC staining of cleaved caspase‐3 in tumor sections of MDA‐MB‐468, MDA‐MB‐231, and patient‐derived xenografts. Scale bar, 100 µm. J) H‐scores of cleaved caspase‐3 staining in tumor sections of MDA‐MB‐468 (*n* = 10), MDA‐MB‐231 (*n* = 12), and patient‐derived xenografts (*n* = 10). Two representative images per tumor were used to quantify cleaved caspase‐3 staining. Data are represented as mean ± SD. *p* values were calculated using one‐way ANOVA, ****p < 0.0001.

To evaluate the efficacy of co‐targeting CDK4/6 and CDK7, we prioritized FDA‐approved drugs or those that have completed clinical trials to enhance clinical relevance. Abemaciclib and palbociclib were chosen as the CDK4/6 inhibitors due to their extensive therapeutic landscape. Using IC50 assays to determine optimal drug concentrations (Figure S2A, Supporting Information), we found that co‐administration of abemaciclib or palbociclib with YKL‐5‐124 profoundly suppressed TNBC clonogenicity (**Figure** **2**A,B). As expected, abemaciclib plus YKL‐5‐124 more effectively inhibited TNBC cell proliferation compared to monotherapy (Figure S2B, Supporting Information). To determine whether these agents worked synergistically rather than additively, we performed a combination index analysis (Figure 2C; Figure S2C, Supporting Information). The results demonstrated broad synergistic effects between abemaciclib and YKL‐5‐124, as well as palbociclib and YKL‐5‐124, across all five TNBC cell lines tested (Figure 2D; Figure S2D, Supporting Information). We also selected CT‐7001 as another CDK7 inhibitor for its improved selectivity and promising results from completed phase I trials.^[^ ^28^ ^]^ The combination of CT‐7001 with abemaciclib also significantly inhibited colony formation (Figure S2E, Supporting Information).

To validate the in vivo synergistic effects, we established cell‐derived xenografts utilizing MDA‐MB‐468 and MDA‐MB‐231 cell lines, as well as a patient‐derived xenograft (PDX). Combination treatments exhibited a notable reduction in tumor weight compared to single‐agent treatment (Figure 2E). Diminished IHC staining intensity of the proliferative marker Ki‐67 verified these antitumor effects (Figure 2F,G). Moreover, co‐administration of abemaciclib and YKL‐5‐124 synergistically induced apoptosis in vitro, evidenced by increased Annexin V‐positive cells (Figure 2H) and upregulated cleaved caspase‐3 staining in xenograft models (Figure 2I,J), validating the enhanced apoptotic response. Taken together, our findings suggest that co‐targeting CDK4/6 and CDK7 synergistically suppresses TNBC proliferation and promotes apoptosis both in vitro and in vivo.

### 2.3 Combination Treatments Suppress SREBP1‐Regulated Cholesterol Synthesis

**Figure 3:**
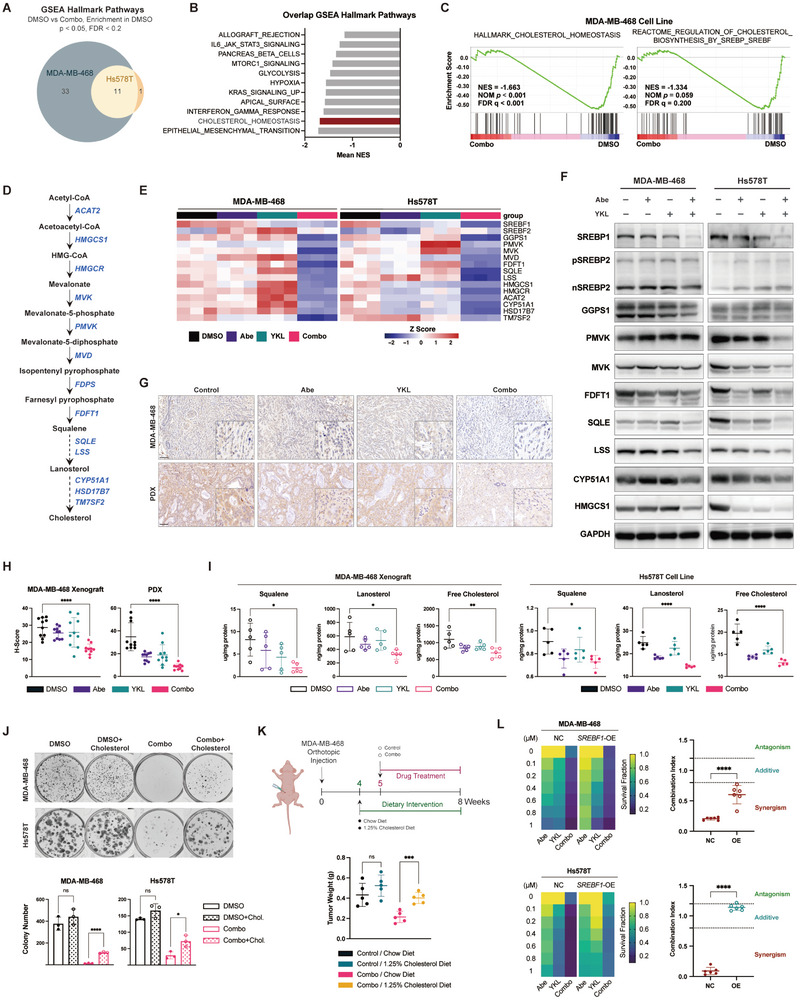
Concurrent inhibition of CDK4/6 and CDK7 suppresses SREBF1‐regulated cholesterol synthesis. A) Venn diagram illustrating the overlapping GSEA hallmark pathways that are enriched in the DMSO groups of MDA‐MB‐468 and Hs578T cells compared to the drug combination (Combo) groups. GSEA was performed using the RNA‐Seq data after 48 h of treatment with DMSO, abemaciclib, YKL‐5‐124, or the combination (*n* = 2 biological replicates). B) The overlapping hallmark pathways ranked by mean NES of MDA‐MB‐468 and Hs578T cells. C) GSEA enrichment plots of cholesterol‐related pathways in the DMSO groups of MDA‐MB‐468 cells. D) Schematic diagram of the cholesterol biosynthesis pathway, including intermediate metabolites (black) and key enzymes (blue). E) Heatmap summarizing the RT‐qPCR results of cholesterol synthesis‐related genes following single‐agent or combined treatment across two TNBC cell lines (*n* = 3). F) Immunoblot analysis of cholesterol synthesis‐related proteins following single‐agent or combined treatment across two TNBC cell lines. G) IHC staining of SREBP1 in tumor sections of MDA‐MB‐468 and patient‐derived xenografts. Scale bar, 100 µm. H) H‐scores of SREBP1 staining in tumor sections of MDA‐MB‐468 (*n* = 10) and patient‐derived xenografts (*n* = 10). Two representative images per tumor were used to quantify SREBP1 staining. Data are represented as mean ± SD. *p* values were calculated using one‐way ANOVA, ****p < 0.0001. I) Quantification of cholesterol‐related metabolites in MDA‐MB‐468 xenografts (*n* = 5) and Hs578T cells (*n* = 5). Data are presented as mean ± SD. *p* values were calculated using one‐way ANOVA, *p < 0.05, **p<0.01, ****p < 0.0001. J) Cholesterol rescued colony formation of combination groups in MDA‐MB‐468 and Hs578T cells. Rescue groups were additionally supplemented with 0.2 µg mL^−1^ cholesterol for 14 days. Representative images from 3 biological replicates are provided. *p* values were calculated using one‐way ANOVA, *p < 0.05, ****p < 0.0001. K) Schematic illustration of the in vivo cholesterol rescue experiment. Mice bearing MDA‐MB‐468 xenograft tumors were randomized to receive a control or a 1.25% cholesterol‐enriched diet, with or without the combination therapy (50 mg kg^−1^ abemaciclib plus 2 mg kg^−1^ YKL‐5‐124). Tumor weights at the study endpoint for the four treatment arms were collected: control with a chow diet, control with a 1.25% cholesterol diet, combined treatments with a chow diet, and combined treatments with a 1.25% cholesterol diet (*n* = 5). Data are shown as mean ± SD. *p* values for tumor weights were determined using a two‐tailed Student's *t*‐test, ***p < 0.001. L) Effects of SREBF1 overexpression (OE) upon drug synergy. Left, heatmaps of viability in SREBF1‐NC and SREBF1‐OE cells after a 48 h exposure to the indicated concentrations of abemaciclib, YKL‐5‐124, and the combined treatment (gradient concentrations of abemaciclib in combination with 1 µM YKL‐5‐124). Data are presented as the mean values from three biological replicates. Right, combination index values for SREBF1‐NC and SREBF1‐OE cells after the combined treatments. Data are shown as mean ± SD. *p* values were determined using an unpaired *t*‐test, ****p < 0.0001.

To investigate the mechanism of synthetic lethality, we performed transcriptional profiling following combination treatments. Given the absence of overlapping hallmark pathways enriched in the combination groups, our focus shifted to the overlapping pathways enriched in the DMSO groups (**Figure** **3**A). Since the top enriched pathway “Epithelial_Mesenchymal_Transition ” has already been investigated in the context of CDK4/6 inhibition,^[^ ^29^ ^]^ our attention was directed toward the unexplored pathway “Cholesterol_Homeostasis” (Figure 3B). Both DMSO groups of MDA‐MB‐468 and Hs578T cells displayed significant enrichment of gene sets associated with cholesterol homeostasis and cholesterol synthesis regulation (Figure 3C; Figure S3A, Supporting Information). The mRNA and protein levels of key enzymes (highlighted in blue, Figure 3D) in the synthetic cascade were markedly decreased with combined treatment (Figure 3E,F). The regulatory axis for these pivotal enzymes is governed by the sterol regulatory element‐binding protein (SREBP) family, primarily comprising SREBP1 and SREBP2 (encoded by SREBF1 and SREBF2, respectively).^[^ ^30^ ^]^ Dual inhibition selectively reduced SREBP1 mRNA and protein levels, while no evident alterations were noted in SREBP2 (Figure 3E,F), indicating the regulatory role primarily attributed to SREBP1. Immunohistochemistry (IHC) of in vivo tumors confirmed the reduction of SREBP1 and enzyme expression observed in vitro (Figure 3G,H; Figure S3B,C, Supporting Information). Metabolomic profiling demonstrated a notable decrease in the intermediate metabolites (squalene, lanosterol) and ultimate products (free cholesterol) of the cholesterol synthesis pathway in cellular and animal models (Figure 3I).

To determine whether suppressed cholesterol synthesis mediated synergistic lethality, we cultured cells with exogenous cholesterol. We found that supplementation of cholesterol and its precursors, squalene and lanosterol, rescued the dual inhibition efficacy with enhanced colony numbers (Figure 3J; Figure S3D, Supporting Information). Similarly, mice fed with a 1.25% high‐cholesterol diet displayed attenuated anti‐tumor effects in vivo, with greater tumor burdens than those on a standard chow diet (Figure 3K). However, additional cholesterol supplementation could not rescue apoptosis levels (Figure S3E, Supporting Information). Furthermore, SREBF1 overexpression impaired combinatorial efficacy, as evidenced by a substantial increase in the combination index and clonogenicity across cell lines (Figure 3L; Figure S3F,G, Supporting Information).

### 2.4 FOXM1 Regulates Cholesterol Metabolism by Directly Binding to SREBF1

**Figure 4:**
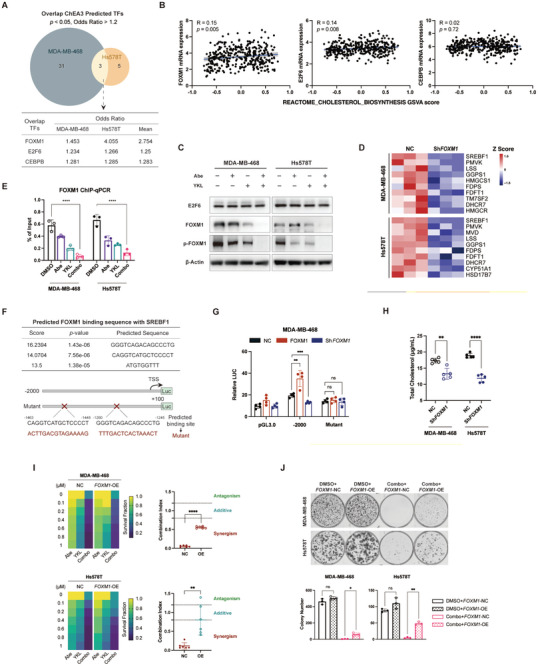
FOXM1 modulates cholesterol homeostasis via directly binding to SREBF1. A) Venn diagram displaying the overlap transcription factors that are predicted from the MDA‐MB‐468 and Hs578T RNA‐Seq data using the ChEA3 database (https://maayanlab.cloud/chea3/). B) Correlation analyses of FOXM1, E2F6, and CEBPB expression with cholesterol synthesis signature scores in the FUSCC‐TNBC cohort (*n* = 360). *p* values were obtained using the Pearson correlation test. C) Immunoblot analysis of E2F6, FOXM1, and p‐FOXM1 (Thr600) in MDA‐MB‐468 and Hs578T cells treated with abemaciclib, YKL‐5‐124, and their combination for 48 h. D) Heatmap showing the RT‐qPCR results of cholesterol synthesis‐related genes after FOXM1 knockdown (*n* = 3). E) FOXM1 ChIP‐qPCR at the promoter regions of SREBF1 following the indicated treatments in MDA‐MB‐468 and Hs578T cells (*n* = 3). Data are presented as mean ± SD. P values were calculated using one‐way ANOVA, ****p < 0.0001. F) Predicted FOXM1 binding sites in the SREBF1 promoter region identified using the hTFtarget database. Mutant SREBF1 promoter sequences are displayed below. G) Analysis of luciferase activity in MDA‐MB‐468 cells (*n* = 4). Data are shown as mean ± SD. P values were calculated using two‐way ANOVA, **p < 0.01, ***p < 0.001. H) Total cholesterol levels in MDA‐MB‐468 and Hs578T cells after FOXM1 knockdown (*n* = 5). The cholesterol concentration was normalized to cell number. Data are shown as mean ± SD. P values were determined using an unpaired *t*‐test, **p < 0.01, ****p < 0.0001. I) Effects of FOXM1 overexpression (OE) upon drug synergy. Left, heatmaps of viability in FOXM1‐NC and FOXM1‐OE cells after a 48 h exposure to the indicated concentrations of abemaciclib, YKL‐5‐124, and the combined treatment (gradient concentrations of abemaciclib in combination with 0.5 µM YKL‐5‐124). Data are presented as the mean values from three biological replicates. Right, combination index values for FOXM1‐NC and FOXM1‐OE cells after the combined treatments. Data are shown as mean ± SD. *p* values were determined using an unpaired *t*‐test, **p < 0.01, ****p < 0.0001. J) FOXM1‐OE‐rescued colony formation of MDA‐MB‐468 and Hs578T cells. Representative images from 3 biological replicates are provided. *p* values were calculated using one‐way ANOVA, *p < 0.05, **p < 0.01.

RNA‐Seq analysis of MDA‐MB‐468 and Hs578T cells identified three potential upstream transcription factors: forkhead box M1 (FOXM1), E2F transcription factor 6 (E2F6), and CCAAT/enhancer binding protein beta (CEBPB) (**Figure** **4**A). Both FOXM1 and E2F6 showed a weak positive correlation with the cholesterol synthesis signature (Figure 4B). Immunoblot results indicated no significant change in E2F6 expression after combination treatment, while total FOXM1 and p‐FOXM1 (Thr600) levels significantly decreased (Figure 4C). Moreover, the reduction in total FOXM1 protein following combined inhibition was time‐dependent (Figure S4A, Supporting Information), implying its potential role as a key regulator for downstream cholesterol biogenesis.

To explore the underlying mechanisms of FOXM1 depletion, we initially assessed FOXM1 mRNA levels and found that co‐administration significantly reduced, but did not completely suppress, FOXM1 RNA levels (Figure S4B, Supporting Information). We therefore hypothesized that the combination treatment not only inhibited FOXM1 transcription but also regulated it post‐transcriptionally. By using cycloheximide (CHX) to exclude the influence of protein synthesis, we observed that FOXM1 degradation occurred in a time‐dependent manner (Figure S4C, Supporting Information). As protein degradation is primarily mediated by the autophagy–lysosome system and the ubiquitin–proteasome system,^[^ ^31^ ^]^ we treated cells with combined treatment plus either autophagy inhibitor 3‐MA, lysosomal inhibitor NH4Cl, or proteasome inhibitor MG132. MG132 partially rescued FOXM1 expression (Figure S4D, Supporting Information), suggesting that FOXM1 may be degraded via the proteasome pathway.

To determine whether FOXM1 directly affects cholesterol synthesis, we constructed FOXM1 knockdown TNBC cells (Figure S4E, Supporting Information) and found that FOXM1 knockdown significantly reduced the expression of SREBF1 and downstream cholesterologenic enzymes (Figure 4D). Therefore, we hypothesized that FOXM1 directly impacts SREBF1, which in turn regulates the expression of downstream key cholesterologenic enzymes. ChIP‐qPCR assays validated FOXM1 occupancy at SREBF1 promoter regions, while combination treatments notably decreased FOXM1 binding (Figure 4E). Putative FOXM1 binding sites were then predicted using the hTFtarget database (Figure 4F). Luciferase reporter assays showed that FOXM1 activated SREBF1 transcription through wild‐type promoters, while mutations markedly blocked FOXM1‐induced transcriptional activities (Figure 4G; Figure S4F, Supporting Information). FOXM1 knockdown impaired SREBF1 regulation on cholesterol synthesis, resulting in decreased total cholesterol levels (Figure 4H). Moreover, FOXM1 overexpression rescued the combinatorial efficacy, as demonstrated by a significant increase in the combination index and clonogenic potential across cell lines (Figure 4I,J; Figure S4G, Supporting Information). Collectively, these findings indicate that FOXM1 promotes cholesterol biogenesis through the transcriptional regulation of SREBF1 expression.

### 2.5 SREBP1 Interacts with p300 to Jointly Govern De Novo Cholesterol Biogenesis

**Figure 5:**
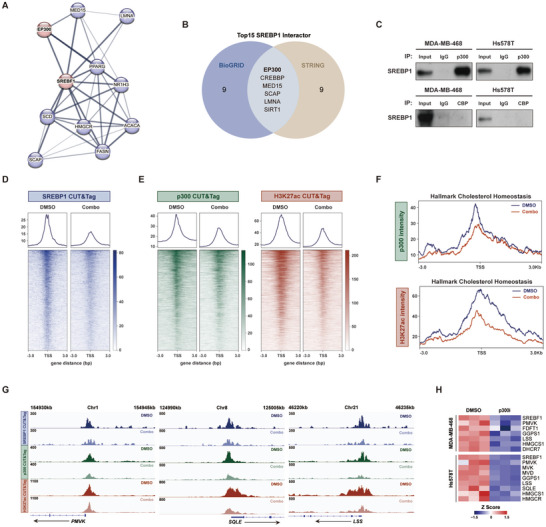
SREBP1 interacts with p300 to collaboratively regulate de novo cholesterol biosynthesis. A) Protein‐protein interaction network centered on SREBF1 as depicted in the STRING database. B) Venn diagram showing the overlapping SREBF1‐interacting proteins identified in the STRING and BioGRID databases. C) Immunoprecipitation followed by immunoblot analysis of SREBP1 and p300/CBP in MDA‐MB‐468 and Hs578T cells. D) Heatmaps visualizing the occupancy of SREBP1 after 72 h of DMSO and combination treatments. Peaks are centered on the transcription start site (TSS) of SREBP1‐bound genes. Data are representative results of two independent experiments. E) Heatmaps visualizing the p300 and H3K27ac signals on SREBP1‐target genes. Data are representative results of two independent experiments. F) P300 and H3K27ac signal intensity at TSS of cholesterol homeostasis genes. Data are representative results of two independent experiments. G) CUT&Tag tracks showing the SREBP1, p300, and H3K27ac signals at the genomic loci of PMVK, SQLE, and LSS. Data are representative results of two independent experiments. H) Heatmap summarizing the RT‐qPCR results of cholesterol synthesis‐related genes after the administration of p300 inhibitors (*n* = 3).

To understand how SREBP1 regulates cholesterol homeostasis following combination therapy, we performed a screening for SREBP1‐interacting proteins using the STRING and BioGRID databases (**Figure** **5**A). This analysis identified the histone acetyltransferases p300 and CREB‐binding protein (CBP) (encoded by EP300 and CREBBP, respectively) as shared candidates for SREBP1 binding partners in both datasets (Figure 5B). Immunoprecipitation demonstrated that p300, but not CBP, physically interacted with SREBP1 in TNBC cells (Figure 5C). CUT&Tag mapping of SREBP1, p300, and H3K27ac (acetylation on histone H3 lysine 27) binding revealed global reductions in SREBP1 occupancy and decreased recruitment of p300 and H3K27ac to SREBP1‐bound genes upon dual inhibition (Figure 5D,E). Moreover, p300 and H3K27ac signal intensities on cholesterol homeostasis genes were significantly reduced after the combined treatments (Figure 5F), as evidenced by decreased promoter occupancy at several cholesterogenic genes, including phosphomevalonate kinase (PMVK), squalene epoxidase (SQLE) and lanosterol synthase (LSS) (Figure 5G). Additionally, p300 blockade with A‐485 or C646 markedly suppressed the transcriptional activities of SREBF1 and essential enzymes involved in cholesterol synthesis (Figure 5H). These results uncovered a cooperative SREBP1‐p300 complex that modulates the cholesterol synthesis pathway.

### 2.6 Clinical Relevance of the SREBP1‐p300‐Cholesterol Synthesis Pathway in TNBC

**Figure 6:**
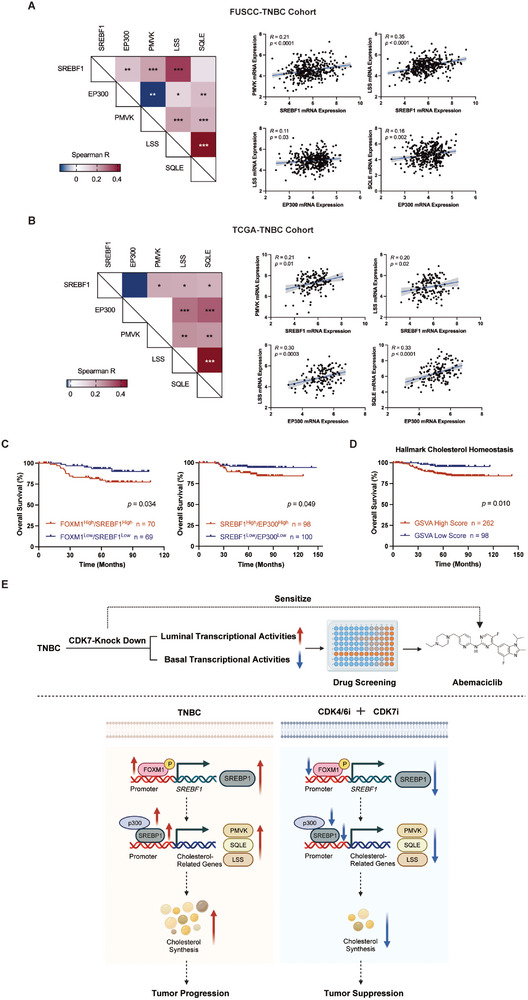
Clinical relevance of the SREBP1‐p300‐cholesterol synthesis pathway in TNBC. A,B) Correlation analysis between the mRNA expression levels of SREBF1, EP300, and cholesterol biosynthesis genes (PMVK, SQLE, and LSS) in the FUSCC‐TNBC (*n* = 360) (A) and TCGA‐TNBC (*n* = 140) (B) cohorts. Correlation coefficients were calculated using the Spearman test. *p* values were obtained using spearman correlation test. C) Kaplan–Meier plots of FOXM1, SREBF1, and EP300 expression in the FUSCC‐TNBC cohort. Data were analyzed using the log‐rank test. D) Kaplan–Meier plots of cholesterol homeostasis gene set variation analysis (GSVA) scores in the FUSCC‐TNBC cohort. Data were analyzed using the log‐rank test. E) Schematic diagram illustrating the proposed mechanism of co‐inhibiting CDK4/6 and CDK7 suppresses SREBP1‐regulated cholesterol biosynthesis. Reduced CDK7 expression disrupts luminal and basal transcriptional activities within TNBC, enabling tumors to overcome intrinsic resistance to CDK4/6 inhibitors. The synergistic intervention initially diminishes the activation of FOXM1, which directly binds with the promoter of SREBF1, exerting regulatory control over SREBF1 transcriptional activity. Consequently, SREBF1 mRNA and protein levels are decreased, attenuating SREBP1/p300 co‐recruitment to cholesterol synthesis gene promoters. This cascade transcriptionally represses rate‐limiting cholesterogenic enzymes, lowers cholesterol synthesis, and maintains antitumor effects.

To investigate the clinical significance of our findings, we conducted a comprehensive analysis of SREBF1, EP300, and cholesterogenic genes (PMVK, SQLE, and LSS) utilizing transcriptomic data from the FUSCC‐TNBC, TCGA‐TNBC, and METABRIC‐TNBC cohorts. We consistently observed weak positive correlations between SREBF1/EP300 and cholesterogenic genes (PMVK, SQLE, and LSS) in three independent TNBC cohorts (**Figure** **6**A,B; Figure S5A, Supporting Information). Moreover, within the FUSCC‐TNBC cohort, patients with high co‐expression of FOXM1/SREBF1 and SREBF1/EP300 exhibited worse OS than those with lower expression levels (Figure 6C). Notably, high cholesterol homeostasis gene signature scores, determined by gene set variation analysis (GSVA), were also strongly associated with poor prognosis in the FUSCC‐TNBC cohort (Figure 6D). Multivariate analysis confirmed that a high cholesterol signature score (hazard ratio = 3.84, *p* = 0.012) was an independent prognostic factor for overall survival in TNBC (Figure S5B, Supporting Information). Collectively, these clinical data from independent TNBC cohorts provide additional support for our experimental findings and proposed mechanisms, linking dysregulated cholesterol metabolism to more aggressive TNBC.

## 3 Discussion

Here, we provide multidimensional evidence showcasing the potential of CDK7 to overcome innate resistance of TNBC toward CDK4/6 inhibitors, paving the way for promising combination therapy. Genome‐wide CRISPR screening using palbociclib‐sensitive and palbociclib‐resistant T47D cells (HR‐positive breast cancer cell line) also identified CDK7 as the top‐ranked essential gene associated with acquired resistance to CDK4/6 inhibition.^[^ ^32^ ^]^ Combined treatments suppressed the activation of FOXM1, consequently impacting the downstream SREBP1‐regulated cholesterol biogenesis pathway (Figure 6E). Moreover, a phase Ib clinical trial revealed that CT7001 not only exhibited favorable tolerability but also achieved a clinical benefit rate of 20% (4/20) in TNBC patients,^[^ ^28^ ^]^ providing robust translational support for our preclinical findings.

The role of cholesterol metabolism in breast cancer has been extensively investigated. The cholesterol metabolite 27‐hydroxycholesterol (27‐HC) stimulates ER‐dependent breast cancer proliferation by acting as the estrogen receptor ligand.^[^ ^33^ ^]^ Additionally, the binding of 27‐HC to the liver X receptor facilitates metastatic processes in breast cancer murine models.^[^ ^33^, ^34^ ^]^ Beyond these growth‐promoting effects, 27‐HC also maintains breast cancer stem cells, induces epithelial‐mesenchymal transition, and enables mammary tumorigenesis.^[^ ^35^ ^]^ Together, these findings reveal broad pro‐oncogenic effects of cholesterol metabolism. Clinical investigations into the interplay between cholesterol levels and breast cancer prognosis demonstrated that cholesterol‐lowering medications, primarily statins, effectively diminish the recurrence risk of HR‐positive breast cancer.^[^ ^36^, ^37^ ^]^ While many studies have been focused on the role of cholesterol in HR‐positive breast cancer, emerging evidence indicates that aberrant cholesterol biogenesis also promotes TNBC progression.^[^ ^38^, ^39^, ^40^ ^]^ Our study proposes that dual inhibition of CDK4/6 and CDK7 suppresses tumor growth by reducing cholesterol synthesis. The role of cholesterol homeostasis in the context of CDK4/6 inhibitors or CDK7 inhibitors has not been previously investigated. Furthermore, our in vitro and in vivo rescue experiments reinforced our findings that replenishing cholesterol attenuated the therapeutic efficacy of combination treatments.

As a multifunctional transcriptional coactivator, p300 regulates various signaling pathways involved in cellular differentiation, homeostasis, and tumorigenesis.^[^ ^41^ ^]^ In HR‐dependent breast cancer, p300 interacts with steroid receptor co‐activator 3 (SRC‐3) and ER to form the ER activation complex, which regulates the binding of estrogen response elements and promotes the expression of ER target genes.^[^ ^42^ ^]^ Moreover, p300/CBP‐mediated acetylation enhances the stability and transcriptional efficacy of the SREBP family by impeding ubiquitin‐dependent degradation.^[^ ^43^, ^44^ ^]^ Here, we propose that p300 collaboratively binds with SREBP1 at the promoters of genes encoding cholesterol synthesis enzymes. Notably, combined therapeutic interventions yield conspicuous reductions in the binding of p300, SREBP1, and H3K27ac (a marker related to active gene transcription) across the genome, consistent with the transcriptional repression of cholesterogenic genes.

In summary, we propose a novel targeted therapeutic strategy for TNBC patients by dual‐inhibiting CDK4/6 and CDK7. Although we hypothesize that the synergistic efficacy could be partly attributed to the attenuation of FOXM1 activation, it remains to be validated whether combinatorial treatment is more effective in TNBC patients with high FOXM1 expression. Therefore, further investigations are warranted to identify potential biomarkers for drug responsiveness, enabling the selection of a more sensitive patient subset.

## 4 Experimental Section

### Cell Lines

MDA‐MB‐231, Hs578T, MDA‐MB‐468, and HEK293T cells were obtained from Cell Bank/Stem Cell Bank, Chinese Academy of Sciences. CAL‐51 and BT‐549 cells were obtained from the American Type Culture Collection. Culture conditions for all cell lines strictly adhered to the provider's recommendations, with the exception of MDA‐MB‐231 and MDA‐MB‐468, which were cultured in DMEM supplemented with 10% fetal bovine serum (FBS). Each TNBC cell line underwent STR authentication, and regular examinations were performed to ensure the absence of mycoplasma contamination.

### Chemicals

The CDK4/6 inhibitors abemaciclib (S7158) and palbociclib (S1116) were from Selleck. The CDK7 inhibitors YKL‐5‐124 (HY‐101257B) and CT7001 (HY‐103712A) were from MedChemExpress. The screening drugs utilized in this study included paclitaxel (S1150), epirubicin (S1223), capecitibine (S1156), letrozole (S1235), tamoxifen (S1972), fulvestrant (S1191), trastuzumab (A2007), lapatinib (S2111), pyrotinib (S8852), olaparib (S1060), and alpelisib (S2814), all obtained from Selleck. For rescue experiments, cholesterol (S4154), squalene (S4862), and lanosterol (S4755) were purchased from Selleck. The p300 inhibitors A‐485 (S8740) and C646 (S7152) were from Selleck. The protein synthesis inhibitor cycloheximide (S7418), autophagy inhibitor 3‐MA (S2767), and proteasome inhibitor MG132 (S2619) were from Selleck. The lysosomal inhibitor NH4Cl (A9434) was from Sigma.

### Mouse Xenograft Models

Animal experiments were performed in accordance with the protocols approved by the Animal Welfare Committee of Fudan University Shanghai Medical College (Protocol Number: 2019FUSCCJS‐226). For combination efficacy evaluation using MDA‐MB‐231 and MDA‐MB‐468 xenografts, 5 million MDA‐MB‐231 cells and 8 million MDA‐MB‐468 cells (suspended in 200 µL volume, culture medium: matrigel = 1:1) were injected into the fourth mammary fat pad of female nude mice (6 weeks old, nu/nu, Shanghai Model Organisms). Once tumors reached 50–75 mm^3^, mice were randomly assigned to four groups in preparation for drug administration. For the TNBC PDX model, 1–2 mm tumor fragments were excised from a previously established NOD/SCID donor mouse (passage 7, NOD.CB17‐Prkdcscid/J, Shanghai Model Organisms). These fragments were then implanted into the mammary fat pad of female nude mice. Upon attaining a tumor size of 125 mm^3^, mice were randomized to treatment groups. For MDA‐MB‐231, MDA‐MB‐468, and patient‐derived xenografts, mice were subjected to the following treatments: control, abemaciclib (50 mg kg^−1^, gavage daily), YKL‐5‐124 (MDA‐MB‐231 xenografts: 5 mg kg^−1^, i.p. daily, MDA‐MB‐468 xenografts and PDX: 2 mg kg^−1^, i.p. daily), and the combination treatment, for either 2 weeks (MDA‐MB‐231 xenografts) or 3 weeks (MDA‐MB‐468 xenografts and PDX).

For in vivo cholesterol rescue experiments using MDA‐MB‐468 xenografts, after the injection of cells and a 4 week period of growth, mice were grouped and subjected to different diet interventions (chow diet and 1.25% cholesterol diet). Once tumors reached 200 mm^3^, mice were further divided into treatment groups, encompassing the control group as well as the combined treatment group receiving 50 mg kg^−1^ abemaciclib (gavage daily) and 2 mg kg^−1^ YKL‐5‐124 (i.p. daily), and were subjected to drug administration for 3 weeks. Dietary interventions were maintained throughout the drug administration phase. Mice were euthanized after the completion of experiments, and tumors were dissected, fixed, and flash frozen for further research.

### Cell Viability Assays and Combination Index Calculation

For the assessment of half‐inhibitory concentration IC50 values, 5000–10000 cells were seeded in a 96‐well plate and treated with the indicated chemicals at different concentrations for 48 h. Cell viability was measured by CCK‐8 assay (Dojindo, CK04) following the manufacturer's instructions. For the combination index analysis, cells were initially exposed to single agents at gradient concentrations. Subsequently, abemaciclib or palbociclib at previous gradient concentrations and YKL‐5‐124 at fixed concentrations (200 nM for CAL‐51 and MDA‐MB‐231, 500 nM for Hs578T and BT‐549, 1 µM for MDA‐MB‐468), were administered. The fixed concentration was determined around IC25 values based on the literature's recommendations.^[^ ^45^ ^]^ CI calculations were generated using CompuSyn software (version 1.0) with the Chou–Talalay method.^[^ ^46^ ^]^

### In Vitro Cell Proliferation and Colony Formation Assays

For proliferation assays, 1500–4000 cells were seeded in a 96‐well plate, and then treated with 0.5 µM abemaciclib and YKL‐5‐124 (50 nM for CAL‐51, Hs578T, and BT‐549, 100 nM for MDA‐MB‐231 and MDA‐MB‐468), either alone or in combination. Cell viability was assessed using CCK‐8 on days 0, 1, 3, and 5.

For colony formation assays, 800–3000 cells were seeded in 6‐well plates and treated with DMSO, 0.5 µM abemaciclib or palbociclib, 50 nM YKL‐5‐124 or CT7001 (20 nM for MDA‐MB‐231, Hs578T, and MDA‐MB‐468, 50 nM for CAL‐51 and BT‐549), and the combination for 48 h. For metabolite rescue colony formation assays, cells were treated with DMSO or 250 nM abemaciclib plus 20 nM YKL‐5‐124 for 48 h. In the rescued groups, additional supplementation of 0.2 µg mL^−1^ cholesterol, 0.5 µg mL^−1^ squalene, or 0.5 µg mL^−1^ lanosterol was administered for a duration of 14 days. After 10–14 days, cells were fixed with 4% paraformaldehyde for 30 min and stained with crystal violet. Colony pictures were captured by Gelcount (Oxford Optronix Ltd.), and colony numbers were quantified by Fiji (version 2.9.0).

### Genetic Construction

Human shRNA lentiviruses targeting CDK7 (GIEL0325417) and FOXM1 (GIEE0370796) were obtained from GeneChem. The sequences of shRNAs used in this study were as follows (5′‐3′): Negative Control shRNA: TTCTCCGAACGTGTCACGT, shCDK7: GCTGTAGAAGTGAGTTTGTAA, shFOXM1: CAGCTGGGATCAAGATTATTA. Human SREBF1 overexpression lentivirus was acquired from GeneChem (GOSL0327553). SREBF1 and FOXM1 cDNAs were respectively inserted into the GV492 (Ubi‐MCS‐3FLAG‐CBh‐gcGFP‐IRES‐puromycin, GeneChem) and GV358 plasmids (Ubi‐MCS‐3FLAG‐SV40‐EGFP‐IRES‐puromycin, GeneChem) for overexpression, while the empty vector was used as a control. The infected cells were subjected to puromycin selection for 7–10 days and validated by immunoblotting.

### Apoptosis Assays

Cells were exposed to DMSO, 0.5 µM abemaciclib, YKL‐5‐124 (0.2 µM for MDA‐MB‐231 and MDA‐MB‐468, 0.5 µM for Hs578T), and the combination for 96 h. FITC Annexin V Apoptosis Detection Kit I (BD biosciences, 556547) was used to detect early/late apoptotic cells. The stained cells were detected by CytoFLEX Flow Cytometer (Beckman Coulter Inc.) and analyzed with FlowJo software (version 10.6.1).

### Immunoblotting and Immunoprecipitation

For the evaluation of cholesterol synthesis pathway‐related proteins, Hs578T cells were exposed to DMSO, 0.5 µM abemaciclib, 0.5 µM YKL‐5‐124, and the combination for 72 h, while MDA‐MB‐468 cells were exposed to DMSO, 0.5 µM abemaciclib, 1 µM YKL‐5‐124, and combination drugs for 48 h. To determine the E2F6, FOXM1, and p‐FOXM1 levels, DMSO, 0.5 µM abemaciclib, YKL‐5‐124 (0.5 µM for Hs578T, 1 µM for MDA‐MB‐468), and the combined treatments were administered for 48 h. For protein degradation‐related experiments, the following drug concentrations were used: CHX at 20 µM, 3‐MA at 5 mM, NH4Cl at 250 µM, and MG132 at 10 µM.

In brief, cells were lysed in mammalian protein extraction reagent (Thermo Scientific, 78501) supplemented with protease inhibitor cocktail (Roche, 04693116001) and phosphatase inhibitor cocktails (Roche, 04906845001). Protein concentrations were quantified using a BCA protein assay kit (Thermo Scientific, 23227). Proteins were separated by SDS‐PAGE and then transferred onto PVDF membranes (Merck, IPVH00010). After blocking, membranes were incubated with specific primary antibodies overnight at 4 °C, followed by 1 h incubation with appropriate HRP‐conjugated secondary antibodies. Protein signals were detected by LumiBest chemiluminescence substrates (ShareBio, SB‐WB011). Detailed information and dilution ratios for the WB antibodies can be found in Table S1 (Supporting Information).

Immunoprecipitation was conducted using a classic magnetic IP/co‐IP kit (Thermo Scientific, 88804) according to the manufacturer's protocol. Cells were lysed in IP lysis/wash buffer, and then incubated with p300 antibody (CST, 54062, 1:200), CBP antibody (CST, 54062, 1:200), or rabbit IgG isotype control (CST, 3900, diluted to the same concentration as p300 or CBP antibody) overnight at 4 °C. After washing, immunoprecipitates were eluted, collected by magnetic beads, and then analyzed by western blotting.

### IHC

Fresh tissues were fixed overnight using 4% paraformaldehyde and then embedded in paraffin for sectioning (4 µm). Paraffin‐embedded sections were deparaffinized in xylene and rehydrated through a graded series of ethanol solutions (100%–95%–70%). For antigen retrieval, slides were exposed to boiled Tris‐EDTA solution (1 mM, pH 9.0). After antigen retrieval, slides were blocked in 5% BSA and incubated with primary antibodies overnight at 4 °C, followed by 1 h incubation with HRP‐conjugated secondary antibodies at room temperature. The antigens were visualized using DAB staining and counterstained with hematoxylin. Slides were dehydrated through a graded series of ethanol solutions (70%–95%–100%) and mounted with coverslips. All slides were scanned by Digital Pathology 120 (KFBIO, KF‐PRO‐120) and viewed by K‐viewer (version 1.7.1.1). Quantification of ki‐67 positivity was performed using the “positive cell detection” function in QuPath (version 0.5.0). H‐scores were calculated according to the formula: 1 × (percentage of weak staining) + 2 × (percentage of moderate staining) + 3 × (percentage of strong staining).^[^ ^47^ ^]^ Staining intensity and extent were determined using the “IHC Profiler” plugin^[^ ^48^ ^]^ in Fiji (version 2.9.0). Image analysis was conducted on two representative images per tumor acquired at 20× magnification. Detailed information and dilution ratios for the IHC antibodies can be found in Table S1 (Supporting Information).

### Real‐Time Quantitative PCR (RT‐qPCR)

The mRNA expression levels of cholesterol synthesis‐related genes were assessed after 72 h treatment of DMSO, 0.5 µM abemaciclib, 0.5 µM YKL‐5‐124, and the combination in Hs578T cells. Similarly, the mRNA levels were evaluated following 48 h treatment of DMSO, 0.5 µM abemaciclib, 1 µM YKL‐5‐124, and the combination in MDA‐MB‐468 cells. To determine the FOXM1 RNA levels, DMSO, 0.5 µM abemaciclib, YKL‐5‐124 (0.5 µM for Hs578T, 1 µM for MDA‐MB‐468), and the combined treatments were administered for 48 h. The RT‐qPCR data for p300 inhibitors were obtained from Hs578T cells after 48 h of exposure to 15 µM C646 and from MDA‐MB‐468 cells after 6 h of exposure to 50 µM A‐485.

Total RNA was extracted using the E.Z.N.A total RNA kit I (Omega, R6834‐01) following the manufacturer's introductions. Cells were lysed in TRK lysis buffer, purified with 70% ethanol, and eluted through top‐speed centrifugation. RNA purity and concentrations were assessed using NanoDrop Lite (Thermo Scientific). One microgram (1 µg) of purified RNA was reverse transcribed into cDNA using the PrimeScript RT master mix (TAKARA, RR036A). RT‐qPCR was performed on a LightCycler 480 system (Roche Diagnostics) using TB green premix Ex Taq II (TAKARA, RR820A). Relative mRNA levels were quantified using the 2 −ΔΔCt method. Specific primers utilized in the qPCR analysis are listed in Table S2 (Supporting Information).

### Chromatin Immunoprecipitation (ChIP)

FOXM1 ChIP‐qPCR assay was conducted in both Hs578T and MDA‐MB‐468 cells after 48 h treatment with DMSO, abemaciclib, YKL‐5‐124, and drug combinations (Hs578T: 0.5 µM abemaciclib, 0.5 µM YKL‐5‐124, MDA‐MB‐468: 0.5 µM abemaciclib, 1 µM YKL‐5‐124). The ChIP experiments were performed using SimpleChIP plus enzymatic chromatin IP kit (CST, 9005). In brief, cells were crosslinked with 1% formaldehyde at room temperature for 10 min and quenched with glycine for 5 min at room temperature. Nuclei pellets were then digested by micrococcal nuclease and sonicated by Bioruptor Pico (Diagenode Diagnostics) for optimal fragmentation. The size of the DNA fragments was confirmed to range from 200 to 900 bp using 1% agarose gel. Digested chromatins were diluted in ChIP buffer, and then incubated with FOXM1 antibody (CST, 20 459, 1:100) overnight at 4 °C. Immunoprecipitates were collected and washed after incubation with protein G magnetic beads. Chromatins were eluted from the antibody/protein G magnetic beads using a thermomixer (1200 rpm, 65 °C, 30 min) and reverse cross‐linked by NaCl and Proteinase K. The purified DNA was quantified by RT‐qPCR analysis with the specific primers mentioned below:

SREBF1(Promoter) Forward: 5′‐TCCCAGCTTGTGATGATCCAG‐3′,

SREBF1(Promoter) Reverse: 5′‐GAAGGAGGAAGCCAGTACCC‐3′.

### Dual‐Luciferase Reporter Assay

Predicted FOXM1 binding sites were identified in the hTFtarget database (http://bioinfo.life.hust.edu.cn/hTFtarget). Lentiviral transduction was employed to generate an empty vector, FOXM1‐overexpression, and ShFOXM1 cells. The constructed cells were then transiently co‐transfected with 200 ng of pGL3.0, pGL3.0‐SREBF1‐promoter, pGL3.0‐SREBF1‐mutant‐promoter reporter plasmids, along with 10 ng Renilla expression plasmids, using jetPRIME transfection reagent (Polyplus, 114–15). Both reporter plasmids and renilla expression plasmids were obtained from GeneChem (GOSE0370793). Firefly and Renilla luciferase activities were assessed using a dual‐luciferase reporter assay system (Promega, E1910) and measured with a SpectraMax iD3 microplate reader (Molecular Devices).

### Quantification of Metabolites in Cholesterol Synthesis

Metabolite quantification in the cholesterol synthesis pathway was conducted both in vitro using Hs578T cells and in vivo using MDA‐MB‐468 xenografts. Hs578T cells were treated with DMSO, 0.5 µM abemaciclib, 0.5 µM YKL‐5‐124, and the combination for 3 days. Xenografts represented tumors from the following treatment arms: control, 50mg kg^−1^ abemaciclib, 2mg kg^−1^ YKL‐5‐124, and the combination treatment. Lipid metabolites involved in the cholesterol synthesis pathway, including squalene, lanosterol, and free cholesterol, were quantified by LC‐MS analysis. Lipid extraction was carried out following the Bligh and Dyer Method^[^ ^49^ ^]^ by homogenizing cells in a chloroform‐methanol system (v/v = 2:1). 1 mL of n‐hexane was added to the lipid extracts containing standard cocktails (Avanti Polar Lipids). After vigorous vortexing and centrifugation, the upper organic phase containing sterols was collected, while the remaining phase was subjected to a second extraction with another 1 mL of n‐hexane. The resulting mixture was dried using a SpeedVac concentrator (Thermo Scientific) under the organic mode. Samples were added pyridine and BSTFA for derivatization. LC‐MS was performed using QTRAP 6500 plus (SCIEX) and the results were normalized to the cell lysate protein levels.

Total cholesterol content in NC and ShFOXM1 cells was assessed using an Amplex red cholesterol assay kit (Invitrogen, A12216). Cell numbers were kept consistent across all samples, and total cholesterol was extracted following the same method as described above. After centrifugation at 14 000 rpm for 10 min, the upper organic phase containing sterols was collected and vacuum dried using a SpeedVac concentrator (Thermo Scientific) under the organic mode. Cholesterol‐containing samples were then re‐dissolved in reaction buffer and incubated with Amplex red reagent, HRP, cholesterol oxidase, and cholesterol esterase at 37 °C for 30 min. Fluorescence signals were measured at Ex/Em = 560/590 nm with a SpectraMax M5 microplate reader (Molecular Devices). Total cholesterol concentration was calculated based on the constructed standard curve.

### RNA‐Seq

Cells were exposed to DMSO, 0.5 µM abemaciclib, YKL‐5‐124 (1 µM for MDA‐MB‐468, 0.5 µM for Hs578T), and the combination for 48 h. RNA extraction and quantification were performed following the methods mentioned above. RNA integrity was assessed using 2100 Bioanalyzer (Agilent Technologies). RNA‐Seq libraries were prepared using the VAHTS Universal V6 RNA‐seq Library Prep Kit (Vazyme, NR604‐01) and sequenced on the Illumina NovaSeq 6000 platform (Illumina Inc.) with paired‐end reads of 150 bp. FASTQ files were processed through fastp^[^ ^50^ ^]^ to eliminate low‐quality reads, and the resulting clean reads were aligned to human reference genome hg38 using HISAT2.^[^ ^51^ ^]^ Gene expression matrix (FPKM value) was generated using the cufflinks pipeline,^[^ ^52^ ^]^ and differential gene expression analysis (threshold: fold change>2 or fold change<0.5, *p*<0.05) was performed using DESeq2.^[^ ^53^ ^]^ For gene set enrichment analysis (GSEA), the GSEA software (version 3.0) was employed, with the permutation type set as “‘gene set”’ and other parameters set as default.

### CUT&Tag

CUT&Tag libraries were prepared using the Hyperactive in situ ChIP Library Prep Kit (pG/pA‐Tn5) from Vazyme (TD901‐01), following the manufacturer's protocol. Briefly, pretreated conA magnetic beads were incubated with cells in a wash buffer at room temperature. ConA‐bound cells were incubated with 2 µg SREBP1 antibody (Santa Cruz, sc13551), 1 µg p300 antibody (CST, 54062), and 1 µg H3K27ac antibody (Abcam, ab4729) at 4 °C overnight. After washing, samples were incubated with secondary antibodies diluted in a wash buffer containing digitonin (used for cell membrane permeation) at room temperature for 1 h. Samples were incubated with pA‐Tn5 transposase in digitonin‐300 buffer at room temperature for 1 h. Subsequently, samples were incubated in a fragmentation buffer at 37 °C for 1 h to allow Tn5 to cut off DNA fragments. The fragmented DNA was then extracted, purified, ligated with P5 and P7 adaptors, and amplified with P5 and P7 primers. The PCR products were evaluated using 2100 Bioanalyzer (Agilent Technologies) for quality control and sequenced on the Illumina NovaSeq 6000 platform (Illumina Inc.) with paired‐end reads of 150 bp. Raw FASTQ data were quality trimmed by fastp^[^ ^50^ ^]^ to obtain clean reads. The clean reads were aligned to the human reference genome hg38 using Bowtie2.^[^ ^54^ ^]^ Peak calling was conducted by SEACR,^[^ ^55^ ^]^ and peak annotations were performed using CHIPseeker.^[^ ^56^ ^]^ Peak distribution of genes of interest was visualized using IGV (version 2.16.0).

### Clinical Samples

The FUSCC‐TNBC cohort with RNA‐Seq data was sourced from Fudan University Shanghai Cancer Center (Sequence Read Archive Dataset: SRP157974). Detailed information on sample collection and data generation could be found in previous literature.^[^ ^57^ ^]^ A total of 360 female patients diagnosed with TNBC from January 2007 to November 2014 were retrospectively analyzed. Follow‐up data were updated until June 2019 and the median follow‐up time was 67.1 months (interquartile range, 53.8–79.9 months). The cutoffs for FOXM1, SREBF1, and EP300 expression, as well as the GSVA score, were determined using the “surv_cutpoint” function from the survminer package. All procedures involving human participants were conducted in accordance with the 1964 Helsinki Declaration. Clinical samples were collected following the protocols approved by the FUSCC Ethics Committee, with each patient providing written informed consent.

### Public Data Analysis

Survival analyses for the KM‐plot basal cohort were performed using the Kaplan–Meier Plotter website (https://kmplot.com/analysis). CRISPR dependency data were extracted from the DepMap Public 22Q1 Public+Score Chronos dataset, while RNA interference (RNAi) dependency data were sourced from the Achilles+DRIVE+Marcotte DEMETER2 dataset. These gene dependency datasets are available from the Broad Institute DepMap portal (https://depmap.org/portal).

### Statistical Analysis

Statistical analysis was conducted using GraphPad Prism (version 9.5.0) and R software (version 4.3.0). Comparisons between the two groups were evaluated using two‐tailed unpaired Student's *t*‐test or one‐way ANOVA, as appropriate. To assess the variance between growth curves and IC50 curves, two‐way ANOVA was employed. Correlation analysis was performed using Pearson's test for normally distributed data, and Spearman's test for non‐normally distributed data. Survival curves were generated through Kaplan–Meier methods and compared using the log‐rank test. The GSVA score was calculated utilizing the FUSCC‐TNBC transcriptome data through the GSVA package in R studio. A two‐sided *p* < 0.05 was considered statistically significant, with *p* values indicated as *p < 0.05, **p < 0.01, ***p < 0.001, ****p < 0.0001.

### Study Approval

Animal experiments were performed in accordance with the protocols approved by the Animal Welfare Committee of Fudan University Shanghai Medical College (Protocol Number: 2019FUSCCJS‐226). For human participants, this study was conducted in accordance with the ethical principles outlined in the Declaration of Helsinki and approved by the Ethics Committee of Fudan University Shanghai Cancer Center (Protocol Number: IRB1612167‐18). Written informed consent was obtained from all participants prior to enrollment.

### Data Availability

FUSCC‐TNBC RNA‐Seq data were available in the Sequence Read Archive (SRP157974). Clinical and RNA‐Seq data of the TCGA cohort were acquired from the UCSC Xena website (https://xenabrowser.net/datapages). Clinical and RNA‐Seq data of the METABRIC cohort were acquired from the cBioPortal (https://www.cbioportal.org). All other raw data generated in this study are available upon request from the corresponding author.

## Conflict of Interest

The authors declare no conflict of interest.

## Author Contributions

Y.Y., J.L., Z.P., and J.M. contributed equally to this work. Y.Y. and J.L. performed all the experiments with the help of J.M. Z.P. analyzed the sequencing data. L.Z., W.S., X.W., X.Z., Z.Z. J.L., X.C., Z.Y., X.M., J.M., Z.Z., Y.J., and Z.S. contributed intellectual input. Y.Y. drafted the manuscript F.X.C., X.Y., and X.G. conceived the project, designed the experiments, and critically reviewed the manuscript. All authors read and approved the final manuscript.

## Supporting information



Supporting Information

Supporting Information

Supporting Information

## Data Availability

The data that support the findings of this study are available from the corresponding author upon reasonable request.
