# Supplementary material for: Dual Inhibition of CDK4/6 and CDK7 Suppresses Triple‐Negative Breast Cancer Progression via Epigenetic Modulation of SREBP1‐Regulated Cholesterol Metabolism
Source: Adv Sci (Weinh). 2024 Dec 10;12(5):2413103. doi: 10.1002/advs.202413103 (PMC11791979; doi:10.1002/advs.202413103)
Supplement: Supplementary file 1 — Supporting Information [file ADVS-12-2413103-s003.pdf]

ADVANCED SCIENCE Open Access

Supporting Information

for *Adv. Sci.*, DOI 10.1002/advs.202413103

Dual Inhibition of CDK4/6 and CDK7 Suppresses Triple-Negative Breast Cancer Progression via Epigenetic Modulation of SREBP1-Regulated Cholesterol Metabolism

Yilan Yang, Jiatao Liao, Zhe Pan, Jin Meng, Li Zhang, Wei Shi, Xiaofang Wang, Xiaomeng Zhang, Zhirui Zhou, Jurui Luo, Xingxing Chen, Zhaozhi Yang, Xin Mei, Jinli Ma, Zhen Zhang, Yi-Zhou Jiang, Zhi-Min Shao, Fei Xavier Chen, Xiaoli Yu* and Xiaomao Guo**

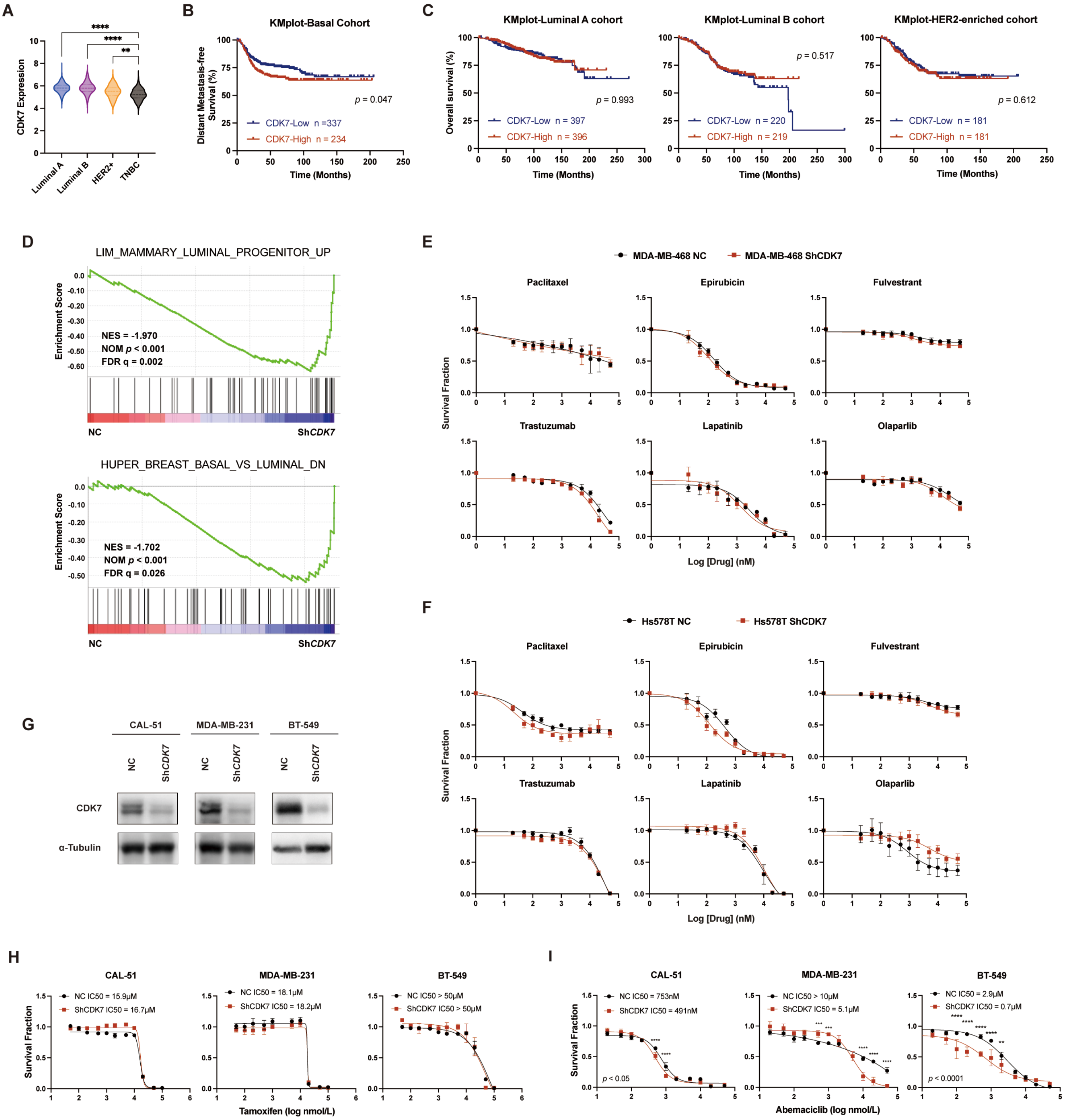
A
CDK7 Expression
Luminal A
Luminal B
HER2+
TNBC
****
**
B
KMplot-Basal Cohort
Distant Metastasis-free Survival (%)
CDK7-Low n =337
CDK7-High n = 234
p = 0.047
Time (Months)
C
KMplot-Luminal A cohort
KMplot-Luminal B cohort
KMplot-HER2-enriched cohort
Overall survival (%)
CDK7-Low n = 397
CDK7-High n = 396
p = 0.993
CDK7-Low n = 220
CDK7-High n = 219
p = 0.517
CDK7-Low n = 181
CDK7-High n = 181
p = 0.612
Time (Months)
D
LIM_MAMMARY_LUMINAL_PROGENITOR_UP
Enrichment Score
NES = -1.970
NOM p < 0.001
FDR q = 0.002
NC
ShCDK7
HUPER_BREAST_BASAL_VS_LUMINAL_DN
NES = -1.702
NOM p < 0.001
FDR q = 0.026
E
MDA-MB-468 NC
MDA-MB-468 ShCDK7
Paclitaxel
Epirubicin
Fulvestrant
Trastuzumab
Lapatinib
Olaparlib
Survival Fraction
Log [Drug] (nM)
F
Hs578T NC
Hs578T ShCDK7
G
CAL-51
MDA-MB-231
BT-549
NC
ShCDK7
CDK7
α-Tubulin
H
NC IC50 = 15.9μM
ShCDK7 IC50 = 16.7μM
NC IC50 = 18.1μM
ShCDK7 IC50 = 18.2μM
NC IC50 > 50μM
ShCDK7 IC50 > 50μM
Tamoxifen (log nmol/L)
I
NC IC50 = 753nM
ShCDK7 IC50 = 491nM
p < 0.05
NC IC50 > 10μM
ShCDK7 IC50 = 5.1μM
NC IC50 = 2.9μM
ShCDK7 IC50 = 0.7μM
p < 0.0001
Abemaciclib (log nmol/L)

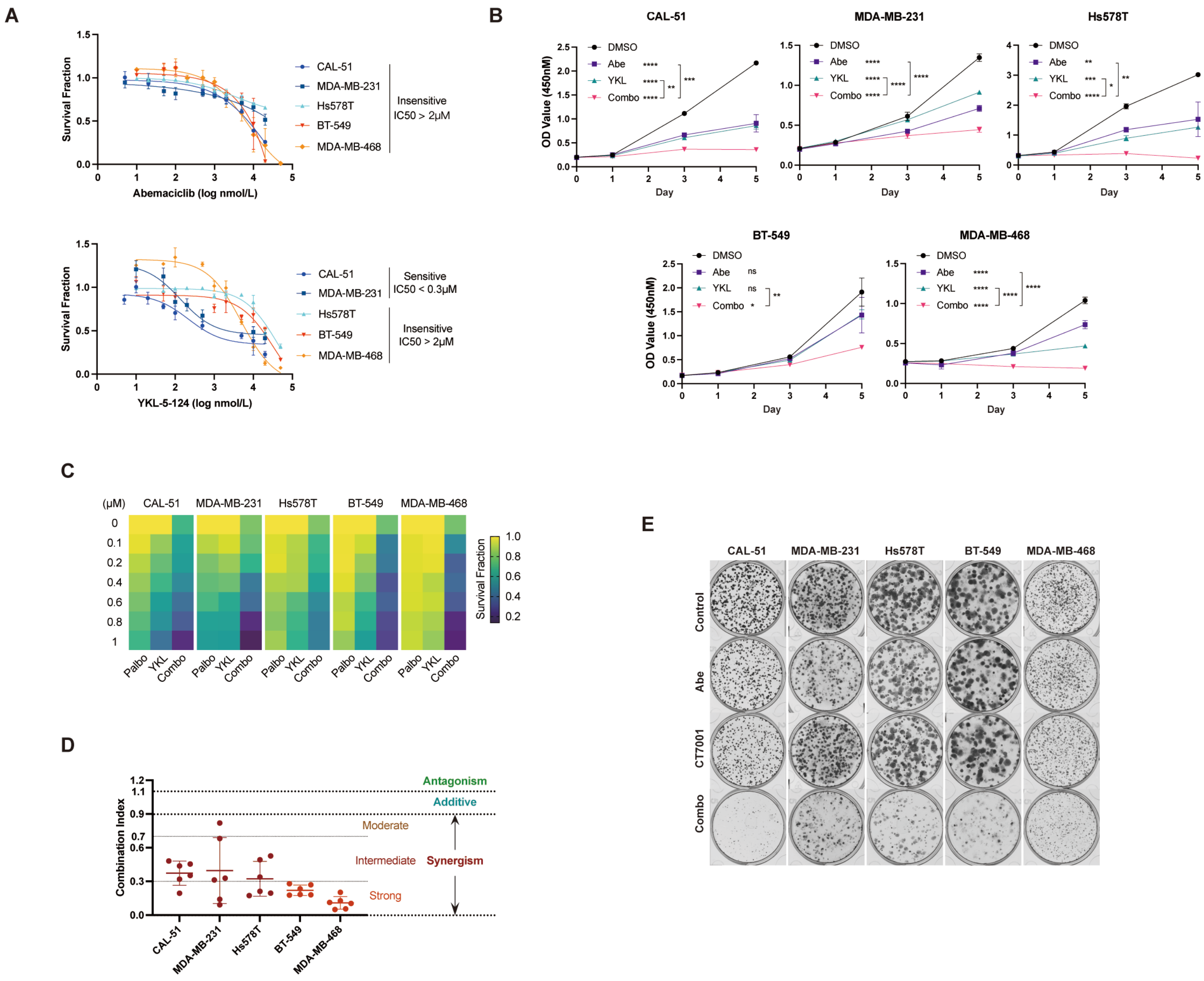
A
Survival Fraction
Abemaciclib (log nmol/L)
CAL-51
MDA-MB-231
Hs578T
BT-549
MDA-MB-468
Insensitive
IC50 > 2μM
YKL-5-124 (log nmol/L)
Sensitive
IC50 < 0.3μM
B
OD Value (450nM)
Day
DMSO
Abe
YKL
Combo
C
(μM)
Palbo
YKL
Combo
Survival Fraction
D
Combination Index
Antagonism
Additive
Moderate
Intermediate
Synergism
Strong
E
Control
Abe
CT7001
Combo

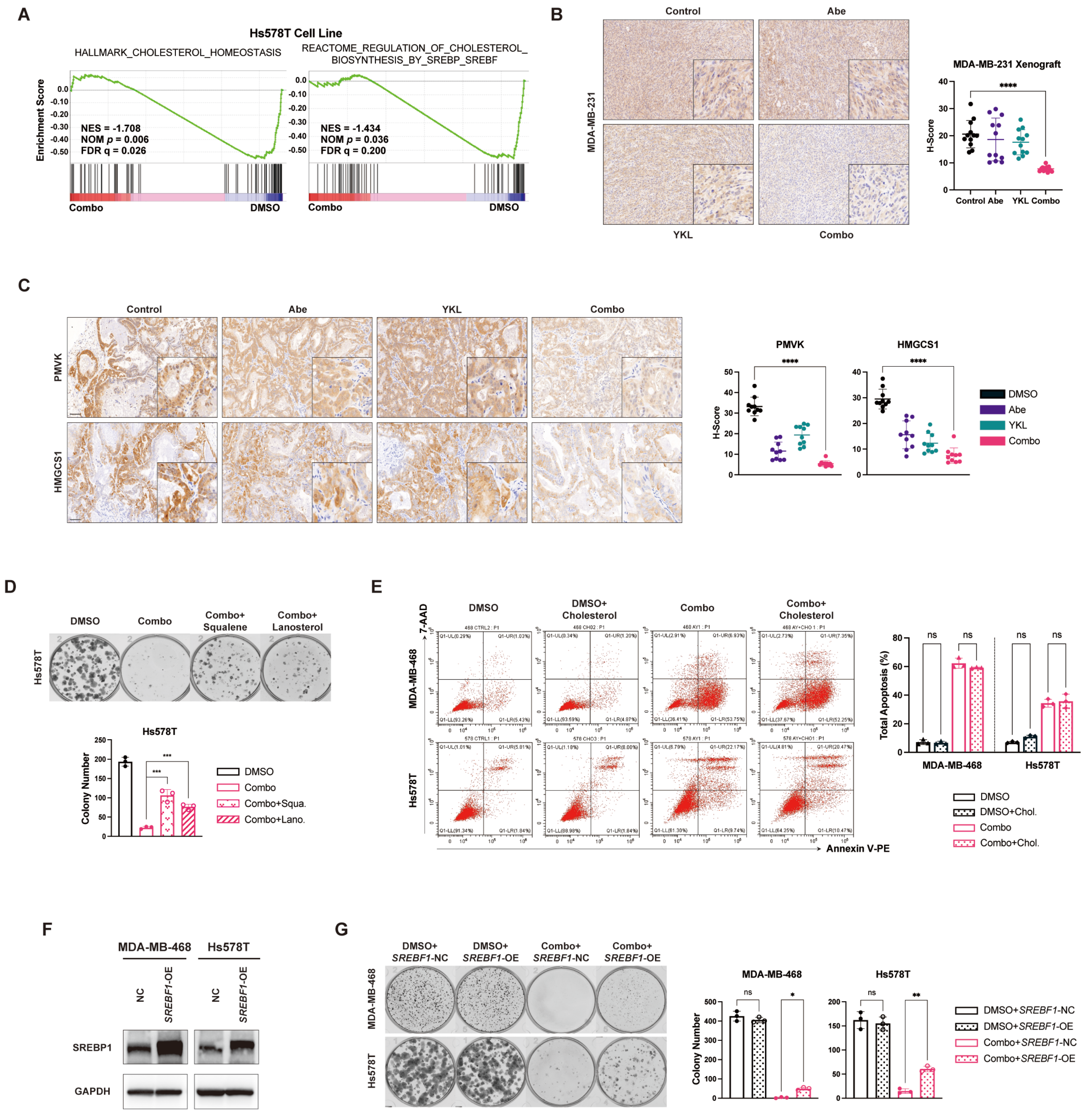

A
Hs578T Cell Line
HALLMARK_CHOLESTEROL_HOMEOSTASIS
REACTOME_REGULATION_OF_CHOLESTEROL_BIOSYNTHESIS_BY_SREBP_SREBF
Enrichment Score
NES = -1.708
NOM p = 0.006
FDR q = 0.026
NES = -1.434
NOM p = 0.036
FDR q = 0.200
Combo
DMSO
B
Control
Abe
YKL
Combo
MDA-MB-231
MDA-MB-231 Xenograft
H-Score
C
PMVK
HMGCS1
DMSO
Abe
YKL
Combo
D
Combo+ Squalene
Combo+ Lanosterol
Hs578T
Colony Number
Combo+Squa.
Combo+Lano.
E
DMSO+ Cholesterol
Combo+ Cholesterol
7-AAD
Annexin V-PE
MDA-MB-468
Total Apoptosis (%)
DMSO+Chol.
Combo+Chol.
F
SREBP1
GAPDH
NC
SREBF1-OE
G
DMSO+ SREBF1-NC
DMSO+ SREBF1-OE
Combo+ SREBF1-NC
Combo+ SREBF1-OE
DMSO+SREBF1-NC
DMSO+SREBF1-OE
Combo+SREBF1-NC
Combo+SREBF1-OE

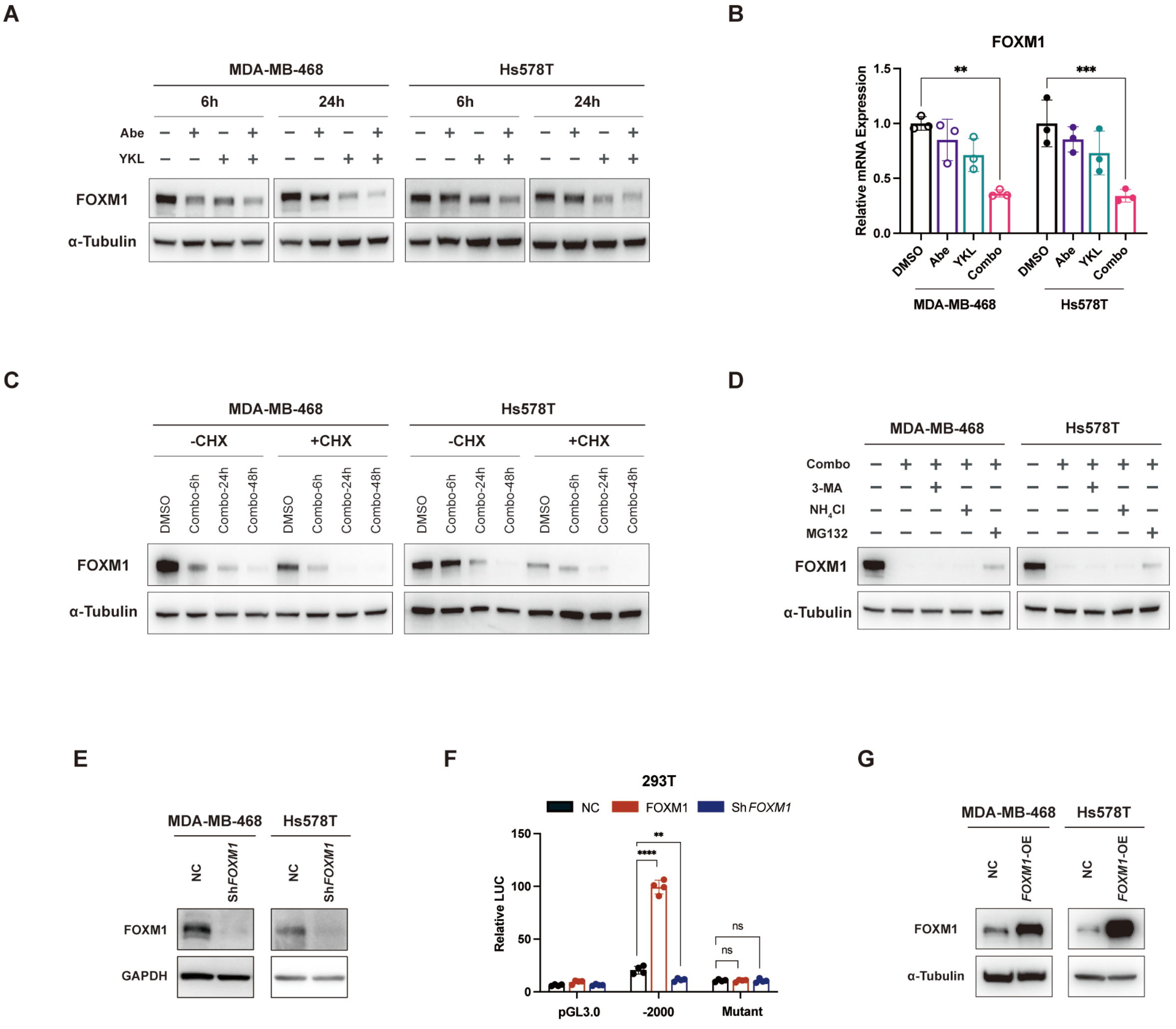
A
MDA-MB-468
Hs578T
6h
24h
6h
24h
Abe
YKL
FOXM1
α-Tubulin
B
FOXM1
Relative mRNA Expression
1.5
1.0
0.5
0.0
**
***
DMSO
Abe
YKL
Combo
MDA-MB-468
Hs578T
C
MDA-MB-468
Hs578T
-CHX
+CHX
DMSO
Combo-6h
Combo-24h
Combo-48h
FOXM1
α-Tubulin
D
MDA-MB-468
Hs578T
Combo
3-MA
NH4Cl
MG132
FOXM1
α-Tubulin
E
MDA-MB-468
Hs578T
NC
ShFOXM1
FOXM1
GAPDH
F
293T
NC
FOXM1
ShFOXM1
Relative LUC
150
100
50
0
****
**
ns
ns
pGL3.0
-2000
Mutant
G
MDA-MB-468
Hs578T
NC
FOXM1-OE
FOXM1
α-Tubulin

A

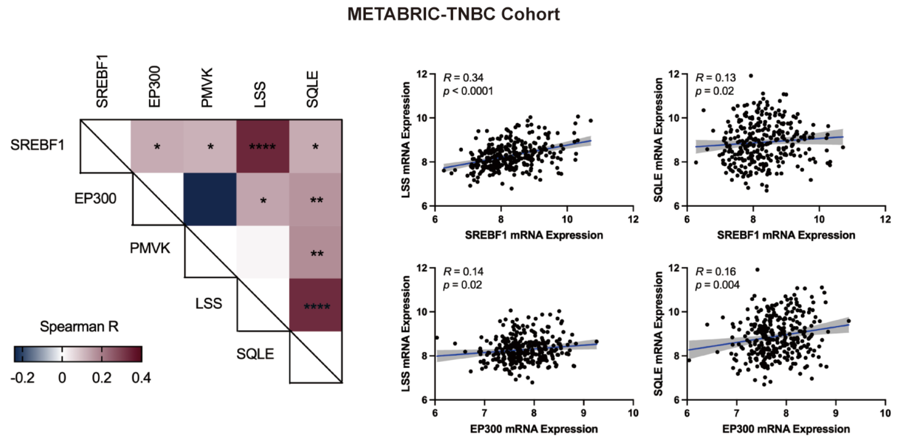

METABRIC-TNBC Cohort
SREBF1
EP300
PMVK
LSS
SQLE
Spearman R
-0.2
0
0.2
0.4
R = 0.34
p < 0.0001
LSS mRNA Expression
SREBF1 mRNA Expression
R = 0.13
p = 0.02
SQLE mRNA Expression
SREBF1 mRNA Expression
R = 0.14
p = 0.02
LSS mRNA Expression
EP300 mRNA Expression
R = 0.16
p = 0.004
SQLE mRNA Expression
EP300 mRNA Expression

B

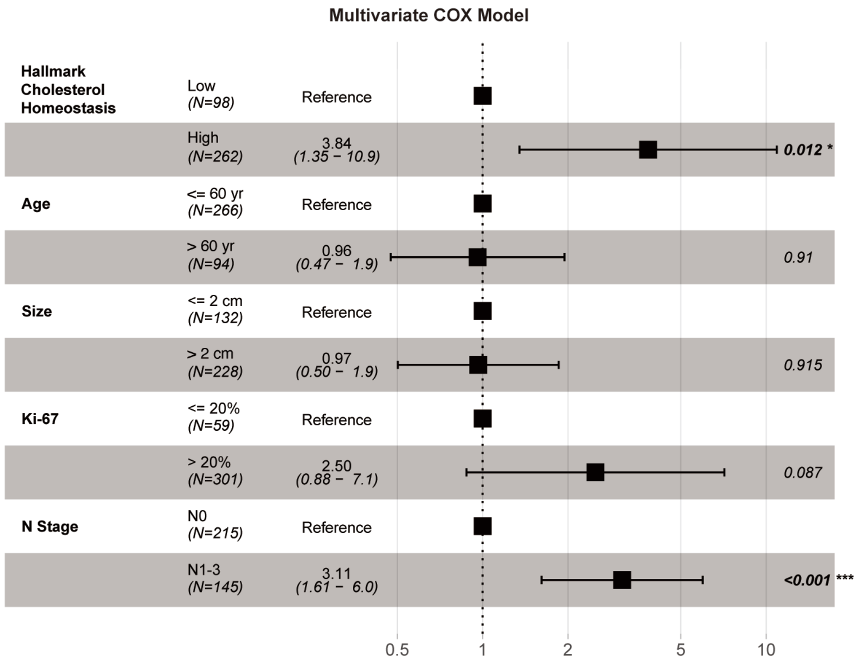

Multivariate COX Model
Hallmark Cholesterol Homeostasis
Low (N=98) Reference
High (N=262) 3.84 (1.35 – 10.9) 0.012 *
Age
<= 60 yr (N=266) Reference
> 60 yr (N=94) 0.96 (0.47 – 1.9) 0.91
Size
<= 2 cm (N=132) Reference
> 2 cm (N=228) 0.97 (0.50 – 1.9) 0.915
Ki-67
<= 20% (N=59) Reference
> 20% (N=301) 2.50 (0.88 – 7.1) 0.087
N Stage
N0 (N=215) Reference
N1-3 (N=145) 3.11 (1.61 – 6.0) <0.001 ***
0.5
1
2
5
10
