# Supplementary material for: Dual Inhibition of CDK4/6 and CDK7 Suppresses Triple‐Negative Breast Cancer Progression via Epigenetic Modulation of SREBP1‐Regulated Cholesterol Metabolism
Source: Adv Sci (Weinh). 2024 Dec 10;12(5):2413103. doi: 10.1002/advs.202413103 (PMC11791979; doi:10.1002/advs.202413103)
Supplement: Supplementary file 2 — Supporting Information [file ADVS-12-2413103-s001.pdf]

ADVANCED SCIENCE
Open Access

Supporting Information

for *Adv. Sci.*, DOI 10.1002/advs.202413103

Dual Inhibition of CDK4/6 and CDK7 Suppresses Triple-Negative Breast Cancer Progression via Epigenetic Modulation of SREBP1-Regulated Cholesterol Metabolism

Yilan Yang, Jiatao Liao, Zhe Pan, Jin Meng, Li Zhang, Wei Shi, Xiaofang Wang, Xiaomeng Zhang, Zhirui Zhou, Jurui Luo, Xingxing Chen, Zhaozhi Yang, Xin Mei, Jinli Ma, Zhen Zhang, Yi-Zhou Jiang, Zhi-Min Shao, Fei Xavier Chen, Xiaoli Yu* and Xiaomao Guo**

Supplementary Tables

Supplementary Table 1. Antibody List

Table S1. Antibody List

Antibodies	Source	Identifier	Application	Dilution
CDK7	CST	2916	WB	1:1000
α-Tubulin	ProteinTech	66031-1-Ig	WB	1:2000
GAPDH	ProteinTech	60004-1-Ig	WB	1:10000
SREBP1	ProteinTech	14088-1-AP	WB	1:1000
SREBP2	ProteinTech	28212-1-AP	WB	1:1000
GGPS1	Abcam	ab167168	WB	1:1000
PMVK	ProteinTech	15674-1-AP	WB	1:1000
MVK	Abcam	ab126619	WB	1:1000
FDFT1	Abcam	ab195046	WB	1:1000
SQLE	ProteinTech	12544-1-AP	WB	1:1000
LSS	Abcam	ab124785	WB	1:50000
CYP51A1	ProteinTech	13431-1-AP	WB	1:1000
HMGCS1	ProteinTech	17643-1-AP	WB	1:1000
E2F6	Abcam	Ab289963	WB	1:1000
FOXM1	CST	5436	WB	1:1000
p-FOXM1 Thr600	CST	14655	WB	1:500
β-Actin	ProteinTech	66009-1-Ig	WB	1:10000
HRP-linked, Anti-Rabbit IgG	CST	7074	WB	1:10000
HRP-linked, Anti-Mouse IgG	CST	7076	WB	1:10000
p300	CST	54062	IP	1:200
CBP	CST	7389	IP	1:200
Rabbit IgG Isotype Control	CST	3900	IP	diluted to the same concentration as p300 or CBP antibody
Ki-67	Abcam	ab15580	IHC	1:1000
Cleaved Caspase-3	CST	9664	IHC	1:400
SREBP1	ProteinTech	14088-1-AP	IHC	1:200
PMVK	ProteinTech	15674-1-AP	IHC	1:200
HMGCS1	ProteinTech	17643-1-AP	IHC	1:200
Goat Anti-Rabbit IgG H&L (HRP)	Abcam	ab205718	IHC	1:2000
Goat Anti-Mouse IgG H&L (HRP)	Abcam	ab205719	IHC	1:2000
FOXM1	CST	20459	ChIP	1:100
SREBP1	Santa Cruz	sc13551	Cut&Tag	—
p300	CST	54062	Cut&Tag	—
H3K27ac	Abcam	ab4729	Cut&Tag	—

Supplementary Table 2. Primer List

Table S2. Primer List

qPCR Primer	Forward Sequence (5'-3')	Reverse Sequence (5'-3')
SREBF1	CGGAACCATCTTGGCAACAGT	CGCTTCTCAATGGCGTTGT
SREBF2	CCTGGGAGACATCGACGAGAT	TGAATGACCGTTGCACTGAAG
GGPS1	ACAGCATCTATGGAATCCCATCT	CAAAAGCTGGCGGGTAAAAAG
PMVK	CCTTTCGGAAGGACATGATCC	TCTCCGTGTGTCACTCACCA
MVK	GGAGCAAGGTGATGTCACAAC	CGGCAGATGGACAGGTATAAGT
MVD	GGACCGGATTTGGCTGAATG	CCCATCCCGTGAGTTCCTC
FDFT1	GCAACGCAGTGTGCATATTTT	CGCCAGTCTGGTTGGTAAAGG
FDPS	TGTGACCGGCAAAATTGGC	GCCCGTTGCAGACACTGAA
SQLE	GGCATTGCCACTTTCACCTAT	GGCCTGAGAGAATATCCGAGAAG
LSS	GTACGAGCCCGGAACATTCTT	CGGCGTAGCAGTAGCTCAT
HMGCS1	GATGTGGGAATTGTTGCCCTT	ATTGTCTCTGTTCCAACTTCCAG
HMGCR	TGATTGACCTTTCCAGAGCAAG	CTAAAATTGCCATTCCACGAGC
ACAT2	GCGGACCATCATAGGTTCCTT	ACTGGCTTGTCTAACAGGATTCT
DHCR7	GCAGGGGTTGTGAACAAGTAT	GAGACGGCATAGCCAAGGAT
CYP51A1	GAAACGCAGACAGTCTCAAGA	ACGCCCATCCTTGTATGTAGC
HSD17B7	TGGGATCATGCCTAATCCACA	CCAGTTCCCGAATCAGGATAAAA
TM7SF2	GTCGCCTGCGCTATCCTATTA	TGCGCCTTCATGTAGAGAAAGA
FOXM1	CGTCGGCCACTGATTCTCAAA	GGCAGGGGATCTCTTAGGTTC
**ChIP-qPCR Primer**	**Forward Sequence (5'-3')**	**Reverse Sequence (5'-3')**
SREBF1(Promoter)	TCCCAGCTTGTGATGATCCAG	GAAGGAGGAAGCCAGTACCC
