# Supplementary material for: Dual Inhibition of CDK4/6 and CDK7 Suppresses Triple‐Negative Breast Cancer Progression via Epigenetic Modulation of SREBP1‐Regulated Cholesterol Metabolism
Source: Adv Sci (Weinh). 2024 Dec 10;12(5):2413103. doi: 10.1002/advs.202413103 (PMC11791979; doi:10.1002/advs.202413103)
Supplement: Supplementary file 3 — Supporting Information [file ADVS-12-2413103-s002.pdf]

ADVANCED SCIENCE
Open Access

Supporting Information

for *Adv. Sci.*, DOI 10.1002/advs.202413103

# Dual Inhibition of CDK4/6 and CDK7 Suppresses Triple-Negative Breast Cancer Progression via Epigenetic Modulation of SREBP1-Regulated Cholesterol Metabolism

*Yilan Yang, Jiatao Liao, Zhe Pan, Jin Meng, Li Zhang, Wei Shi, Xiaofang Wang, Xiaomeng Zhang, Zhirui Zhou, Jurui Luo, Xingxing Chen, Zhaozhi Yang, Xin Mei, Jinli Ma, Zhen Zhang, Yi-Zhou Jiang, Zhi-Min Shao, Fei Xavier Chen*, Xiaoli Yu* and Xiaomao Guo**

## Supplementary Figure Legends

**Figure S1. CDK7-knockdown TNBC cell lines exhibit dysregulation of luminal and basal transcriptional activities, conferring increased sensitivity to abemaciclib, related to Figure 1**

(A) Violin plots depicting CDK7 mRNA expression across luminal A (n=420), luminal B (n=192), HER2-enriched (n=67), and TNBC (n=140) subtypes using data from the TCGA-BRCA dataset. *P* values were calculated using one-way ANOVA, **$p$<0.01, ****$p$<0.0001.

(B) Kaplan-Meier plots of distant metastasis-free survival stratified by CDK7 expression level in the KMplot-Basal cohort. Data were analyzed using the log-rank test.

(C) Kaplan-Meier curves for CDK7 expression in luminal A, luminal B, and HER2-enriched patients form KMplot cohorts. Data were analyzed using the log-rank test.

(D) GSEA plots of RNA-Seq data for normal control (NC) and CDK7-knockdown (ShCDK7) Hs578T cells (n = 3).

(E-F) Representative dose-response curves of drug sensitivities between MDA-MB-468-NC and MDA-MB-468-ShCDK7 cells (E), as well as between Hs578T-NC and Hs578T-ShCDK7 cells (F). Data are mean ± SD of 5 replicates.

(G) Immunoblot validation of CDK7 knockdown in CAL-51, MDA-MB-231, and BT-549 cells.

(H-I) Dose-response curves of tamoxifen (H) and abemaciclib (I) between NC and ShCDK7 CAL-51, MDA-MB-231, and BT-549 cells. Data are mean ± SD of 3-5 experimental replicates. *P* values were analyzed using two-way ANOVA test with Bonferroni correction.

**Figure S2. Dual inhibiting CDK4/6 and CDK7 reduces cell proliferation, related to Figure 2**

(A) Dose-response curves for abemaciclib and YKL-5-124 in TNBC cells. Data are mean ± SD of 3-5 experimental replicates.

(B) In vitro growth curves of TNBC cells after abemaciclib, YKL-5-124, or their combination (n = 3). Data are presented as mean ± SD. *P* values were calculated using two-way ANOVA test, *$p$<0.05, **$p$<0.01, ***$p$<0.001, ****$p$<0.0001.

(C) Heatmap of survival fractions in TNBC cells after 48 hours exposure to gradient concentrations of palbociclib, YKL-5-124, and the combined treatment (palbociclib at gradient concentrations with YKL-5-124 at fixed concentrations). Data are shown as mean (n = 3 biological replicates).

(D) Combination index values for TNBC cells treated with palbociclib plus YKL-5-124, calculated by CompuSyn software. Data are represented as mean ± SD.

(E) Colony formation images of TNBC cells following a 48-hour exposure to the combination of abemaciclib with CT7001. Representative images from 3 biological replicates are provided.

**Figure S3. Combination treatments weaken cholesterol biosynthesis regulated by SREBP1, related to Figure 3**

(A) GSEA enrichment plots of cholesterol-related pathways in the DMSO groups of Hs578T cells.

(B) IHC staining and quantification of SREBP1 in tumor sections of MDA-MB-231 xenografts (n=12). Two representative images per tumor were used to calculate H-scores. Scale bar, 100 μm. Data are represented as mean ± SD. *P* values were calculated using one-way ANOVA, ****$p$<0.0001.

(C) IHC staining and quantification of PMVK and HMGCS1 in tumor sections of PDX (n=10). Two representative images per tumor were used to calculate H-scores. Scale bar, 100 μm. Data are represented as mean ± SD. *P* values were calculated using one-way ANOVA, ****$p$<0.0001.

(D) Squalene and lanosterol rescued colony formation of Hs578T cells. Rescue groups were additionally supplemented with 0.5 μg/mL squalene or 0.5 μg/mL lanosterol for 14 days. Representative images from 3 biological replicates are provided. *P* values were calculated using one-way ANOVA, ***$p$<0.001.

(E) Apoptosis analysis of MDA-MB-468 and Hs578T cells treated with DMSO, DMSO plus cholesterol, combo, and combo plus cholesterol (n=3). The left panel shows representative images from three biological replicates, and the right panel displays the proportion of total apoptotic cells.

(F) Immunoblot validation of SREBP1 overexpression in MDA-MB-468 and Hs578T cells.

(G) SREBF1 overexpression-rescued colony formation of MDA-MB-468 and Hs578T cells. Representative images from 3 biological replicates are provided. *P* values were calculated using one-way ANOVA, *$p$<0.05, **$p$<0.01.

**Figure S4. FOXM1 stimulates the transcriptional activities of SREBF1, related to Figure 4**

(A) Immunoblot analysis of FOXM1 in MDA-MB-468 and Hs578T cells treated with abemaciclib, YKL-5-124, and their combination for 6 and 24 hours.

(B) RT-qPCR results of FOXM1 in MDA-MB-468 and Hs578T cells treated with abemaciclib, YKL-5-124, and their combination for 48 hours (n=3). *P* values were obtained using one-way ANOVA, **$p$<0.01, ***$p$<0.001.

(C) Immunoblot analysis of FOXM1 in MDA-MB-468 and Hs578T cells treated with the combined treatment, with or without cycloheximide (CHX), for the indicated hours.

(D) Immunoblot analysis of FOXM1 in MDA-MB-468 and Hs578T cells treated with the combined treatment for 48 hours plus either autophagy inhibitor 3-MA, lysosomal inhibitor $NH_4Cl$, or proteasome inhibitor MG132.

(E) Immunoblot validation of FOXM1 knockdown in MDA-MB-468 and Hs578T cells.

(F) Analysis of luciferase activity in HEK293T cells (n = 4). Data are shown as mean ± SD. *P* values were determined using two-way ANOVA, **$p$<0.01, ****$p$<0.0001.

(G) Immunoblot validation of FOXM1 overexpression in MDA-MB-468 and Hs578T cells.

**Figure S5. Clinical relevance of the SREBP1-p300-cholesterol synthesis axis in TNBC, related to Figure 6**

(A) Correlation analysis between the mRNA expression levels of SREBF1, EP300, and cholesterol biosynthesis genes (PMVK, SQLE, and LSS) in the METABRIC-TNBC cohort (n=309). Correlation coefficients were calculated using the Spearman test. *P* values were determined using spearman correlation test.

(B) Multivariate COX analysis of overall survival in the FUSCC-TNBC cohort, with Hallmark Cholesterol Homeostasis grouping consistent with Figure 6D.
